# Review: Sex-Specific Aspects in the Bariatric Treatment of Severely Obese Women

**DOI:** 10.3390/ijerph17082734

**Published:** 2020-04-15

**Authors:** Pia Jäger, Annina Wolicki, Johannes Spohnholz, Metin Senkal

**Affiliations:** 1 Department of General and Visceral Surgery, Marien Hospital Witten, Teaching hospital of the Ruhr-University Bochum, Marienplatz 2, 58452 Witten, Germany; 2 Department of General and Visceral Surgery, Marien Hospital Herne, University hospital of the Ruhr-University Bochum, Hölkeskampring 40, 44625 Herne, Germany

**Keywords:** bariatric surgery, obesity, sex-specific treatment, women/females, bariatric aftercare

## Abstract

This systematic literature review aims to point out sex-specific special features that are important in the bariatric treatment of women suffering from severe obesity. A systematic literature search was carried out according to Cochrane and Preferred Reporting Items for Systematic review and Meta-Analysis Protocols (PRISMA-P) guidelines. After the literature selection, the following categories were determined: sexuality and sexual function; contraception; fertility; sex hormones and polycystic ovary syndrome; menopause and osteoporosis; pregnancy and breastfeeding; pelvic floor disorders and urinary incontinence; female-specific cancer; and metabolism, outcome, and quality of life. For each category, the current status of research is illuminated and implications for bariatric treatment are determined. A summary that includes key messages is given for each subsection. An overall result of this paper is an understanding that sex-specific risks that follow or result from bariatric surgery should be considered more in aftercare. In order to increase the evidence, further research focusing on sex-specific differences in the outcome of bariatric surgery and promising treatment approaches to female-specific diseases is needed. Nevertheless, bariatric surgery shows good potential in the treatment of sex-specific aspects for severely obese women that goes far beyond mere weight loss and reduction of metabolic risks.

## 1. Introduction

The prevalence of obesity has increased in the last decades. The World Health Organization (WHO) reports that “obesity is one of today’s most blatantly visible—yet most neglected—public health problems.” With the neologism “globesity,” the WHO describes the increasing prevalence of overweight and obesity in many parts of the world as a global epidemic [1].

In 2016, about 39% of adults (more than 1.9 billion) were overweight and 18% of people (659 million) were obese globally. Therefore, the prevalence tripled from 1975. Among minors and children, the prevalence also increased significantly: in 1975, 4% of children and adolescents ages 5–19 were overweight, and in 2016 18% of girls and 19% of boys were. In the same period, the prevalence of obesity increased from 1% to 6% in girls and 8% in boys [2,3].

For classification of weight, overweight, and obesity, usually the body mass index (BMI; kg/m^2^) is used. This classification is criticized because of the low sensitivity, large interindividual variability in relative body fat, and its attribution to age, sex, and ethnicity. Nevertheless, BMI is the most commonly used instrument in epidemiology and clinical practice due to its simplicity [4,5].

Overweight in adults is defined as a BMI of more than 25 kg/m^2^, and obesity is indicated by a BMI of 30 kg/m^2^. Children and adolescents are overweight if their BMI is more than one standard deviation higher than the reference BMI and obese if their BMI deviates by more than two standard deviations.

Depending on the database, rates of overweight are similar in men and women or slightly higher in men. Obesity occurs more often in women [1,2,6]. Although the main causes, hypercaloric nutrition and physical inactivity, affect both men and women, there are differences in the development and in symptoms and associated comorbidities depending on sex [7]. Regarding treatment and therapeutic approaches, sex-specific aspects must be taken into consideration, too. Overweight and obesity are major risks for a number of diseases and comorbidities, including diabetes mellitus, cardiovascular diseases, cancer, and musculoskeletal diseases [8].

As therapy, conservative and surgical procedures are available. Due to both the increased prevalence of obesity and the available evidence for bariatric surgery, the number of bariatric therapies has increased in the last two decades. Especially in morbidly or severely obese people, bariatric procedures show significantly better results than conservative therapies. An increasing number of national therapeutic guidelines for the treatment of obesity and metabolic diseases recommend bariatric surgery after conservative weight loss or even if initial treatment fails, depending on BMI and comorbid diseases [9,10,11].

Effective bariatric procedures are sleeve gastrectomy, Roux-en-Y gastric bypass, omega-loop gastric bypass, and biliopancreatic diversion with or without duodenal switch. Currently, Roux-en-Y gastric bypass and sleeve gastrectomy are the most commonly performed procedures [10,11].

Traditionally, restrictive bariatric operations are differentiated from malabsorptive procedures. While primary restrictive procedures such as sleeve gastrectomy induce weight loss mostly as a result of restricted dietary intake, malabsorptive procedures such as biliopancreatic diversion are mostly based on restricted nutrient absorption. However, a clear separation is not possible because primary restrictive procedures also benefit from complex, systemic mechanisms such as hormonal changes. Roux-en-Y gastric bypass relies on both restrictive and malabsorptive effects as well as their interaction and is therefore classified as an intermediate procedure. Currently, the proximal Roux-en-Y gastric bypass is the gold standard and globally the most commonly performed procedure, although the number of sleeve gastrectomies exceeded the number of Roux-en-Y gastric bypass surgeries in some European countries [9,11].

**Figure 1:**
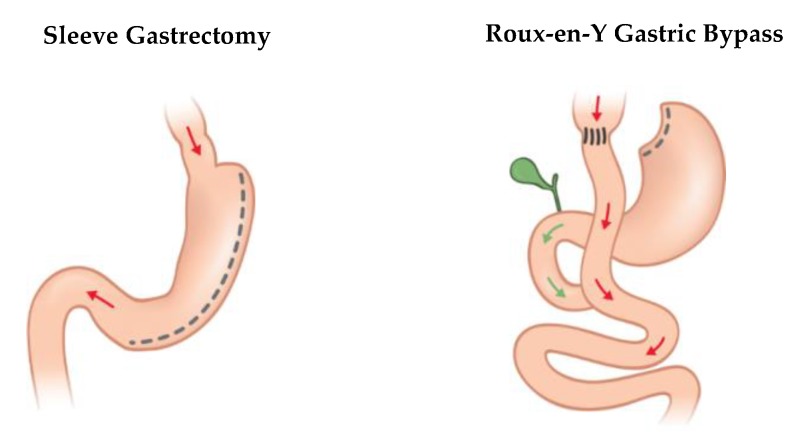
Sleeve gastrectomy and Roux-en-Y gastric bypass (with kind approval of Johnson & Johnson^®^).

Figure 1 gives a graphical overview of the sleeve gastrectomy and Roux-en-Y gastric bypass surgical procedures.

The aim of this paper is to identify sex-specific aspects in the bariatric treatment of women suffering morbid obesity. Based on these aspects, the implications in terms of clinical treatment, recommendations, and key messages for therapeutic practice are worked out.

## 2. Methods

In order to identify current relevant topics, a systematic search was carried out according to the requirements of Cochrane [12] and PRISMA-P [13,14].

### 2.1. Electronic Literature Search in Scientific Databases

A systematic literature search including a number of medical databases of scientific publications for the last 5 years was performed with several online search engines. An overview of the databases and search engines used is presented in Table 1.

Table 2 gives an overview of the results of the literature search. Due to specific features of the search engines, the search functions differed. The search terms in the table are presented in a uniform format with “AND” as a mandatory additional search term and “OR” as an alternative search term. The latter always refers to the search term that was mentioned directly before.

### 2.2. Selection and Inclusion Criteria

The literature search included publications from the last 5 years as of the time of writing and was performed in English. Articles published in English or with an English summary or abstract were taken into consideration. Journal literature, which means original research articles, case reports, systematic reviews, and comments as well as abstracts of recently published posters of peer-reviewed journals, was considered in the systematic literature search and checked for eligibility.

All original research articles and systematic reviews were included. Educational papers were included and named as such. Comments, corrections, and letters to the editor were taken into consideration too if they referred to a research article or review that met the inclusion criteria and were included in this review. Comments that did not refer to recently published articles and did not offer additional scientific value were excluded. In individual cases, abstracts of recently published posters were included if the survey met the scientific criteria.

All studies that focused specifically on the bariatric treatment of women and on sex- or gender-specific aspects were included. For this systematic literature review, no systematic assessment of potential bias was performed. Rather, this paper aims to consider surveys that might be relevant for clinical practice but do not fit the criteria of a meta-analysis, such as case reports. Therefore, no studies were excluded due to potential bias, such as a limited number of cases. If limited evidence was observed, it was named and discussed collectively with the concerning publication. Publications with a clear conflict of interest or insufficient scientific procedures were excluded.

Studies that only dealt with men or children or did not consider sex-specific aspects were excluded. If studies were carried out with a female survey group, e.g., in order to create greater homogeneity, they were carefully verified as to whether gender- or sex-specific aspects were the focus. If this was not the case, the surveys were not taken into consideration.

**Figure 2:**
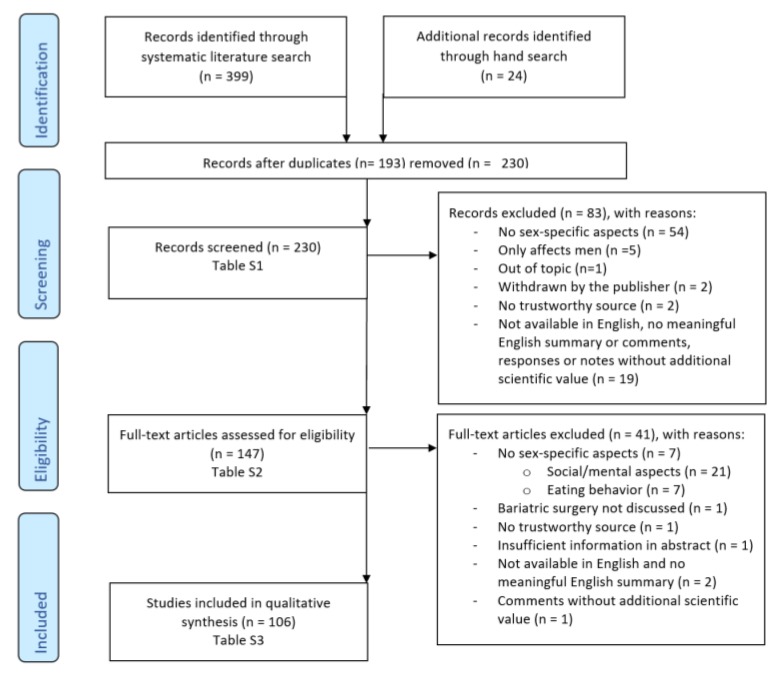
Flow diagram of literature selection.

As a first step, the records were screened after duplicates were removed. The manuscripts that already stood out in the screening because they did not meet the inclusion criteria were already sorted out at this point. The detailed reasons are described in Figure 2. In the next step, the screened results were verified for full-text eligibility and assigned to main categories, as described above. Articles that did not meet the described inclusion criteria on closer examination were excluded during this process. Articles that were not open access but did not offer an abstract with sufficient information to justify buying the article were excluded. The same was true for articles published in languages other than English and without a meaningful English abstract or summary. The reasons for exclusion are described in detail.

After thorough examination of the selected literature, the categories social and mental health aspects and eating behavior and disorders were not included in this review paper. Although some publications in these fields discuss sex-specific aspects, especially in terms of development and prevalence, there were no resulting sex-specific implications in the treatment. Therefore, the criteria regarding sex-specific aspects according to the definition in the Methods section were not met and the articles did not fit the purpose of this review paper focused on treatment.

In this paper, sex-specific aspects are defined as aspects that are associated with sex-specific divergence, for example due to female anatomy or metabolism. In order to provide an overview and recommendations for therapists, this research only focuses on those aspects that result in sex-specific implications.

### 2.3. Summarizing into Main Categories

Based on the publications that were considered for inclusion by screening the results of the systematic literature review, the main categories were developed deductively. If a publication covered topics of more than one main criterion, it was assigned to more than one category.

The results for each category are presented as follows. For articles that met the inclusion criteria but focused on topics for which so few studies are available that a separate category was not possible, the category “other” was defined. All included publications were assigned to these categories. For each category, the results are worked out and summarized. The complete literature search with the process of inclusion and development of the main categories is presented in Table S1. The results of the assignment of the relevant literature into the listed categories are presented in Table S2. Additional information concerning the included publications is summarized in Table S3.

## 3. Results

The literature search and the process of selection of relevant literature are presented in a flow diagram (Figure 2) according to the PRISMA guidelines [13]. An overview of the inclusion criteria and the reasons for exclusion are presented in Table S4 in the Supplementary Materials.

According to the process of category development, which is described in Section 2.3, the following categories were defined:

- Female sexuality and sexual function
- Contraception
- Fertility
- Pregnancy and breastfeeding
- Sexual hormones and polycystic ovary syndrome (PCOS)
- Menopause and osteoporosis
- Pelvic floor disorders and urinary incontinence
- Female-specific cancer
- Metabolism, outcome, and quality of life
- Other
- Social and mental health aspects
- Eating behavior and disorders

### 3.1. Female Sexuality and Sexual Function

The presence of obesity is correlated with sexual and erectile dysfunction. In women, this correlation links obesity to disorders of the female cycle and hormone regulation, which are in turn associated with sexual function. Poor self-acceptance of body image, comorbid mental disorders, and difficulties in interpersonal relationships may aggravate the situation [15].

A widely used standardized instrument for assessing sexual function among women is the Female Sexual Function Index (FSFI). This instrument was developed as a measure of female sexual dysfunction and contains six domains: desire, arousal, lubrication, orgasm, satisfaction, and pain. There is an overall FSFI score, and each domain can be evaluated on its own. Female sexual dysfunction was defined by the developers of the index as an overall score ≤26.55. However, this cutoff is used inconsistently in the scientific literature; for example, a total FSFI score ≤26.55 or ≤23 has been cited. Slight deviations due to language and culturally sensitive validated versions must be considered in this context too [16,17,18].

In a multicenter study, Steffen et al. found limitations in sexual activity due to physical health in 38% and dissatisfaction with their sexual life in 49% of 1751 women surveyed before bariatric surgery [19]. One year after bariatric surgery, 56.0% (95% confidence interval (CI): 52.5%–59.5%) of the women who were dissatisfied with their sexual life reported meaningful improvement. This betterment persisted in follow-up after five years. At this time, 73.6% (95% CI: 69.3%–78.0%) of the women who had physical limitations to sexual activity before bariatric surgery still reported an improvement. The improvement was independent of the type of surgical procedure [20].

Similar results were found by researchers in Brazil. In a survey of 62 women who underwent bariatric surgery, the prevalence of sexual dysfunction decreased from 62% before to 19% six months after treatment. This improvement was found in both overall FSFI score and all included domains. The same authors also found amelioration of several sex positions. As a result, the surveyed women reported adopting a greater variety of sexual positions six months after bariatric surgery [21].

In contrast, a smaller American survey of 106 women found an improvement in total FSFI scores as well as the arousal, desires, and satisfaction domains within the first three years but no more in the fourth year. The authors assumed that there are typical trajectories of sexual function after bariatric surgery that may depend on the initial FSFI score [22]. Nevertheless, the low number of cases must be kept in mind when describing these trajectories.

Two follow-up surveys of 60 and 43 women in the Czech Republic and France, respectively, showed amelioration in all FSFI domains 12 months after bariatric surgery, although in the Czech survey it was only significant regarding the desire category [15,23]. Nevertheless, the scores of the surveyed women remained significantly lower than those of the non-obese female reference group [15]. These results concur with those of Janik et al., who found significantly higher FSFI scores in the desire and arousal domains for 153 women 12–18 months after bariatric surgery. Although the other FSFI domains and the prevalence of female sexual dysfunction did not change significantly, sexual quality of life was significantly higher in follow-up compared to preoperative values [16].

A survey in Malaysia with 52 women found a decrease in sexual dysfunction from 75% before to 36% six months after bariatric surgery. The improvement was significant in all six FSFI categories and correlated with a reduction of BMI [24]. In turn, women with sexual dysfunction, as assessed by the FSFI, showed higher concerns with weight and shape, more depressive symptoms, and more regaining of weight [25].

While sexual function can be improved by weight loss and better physical health, the large proportion of women suffering from excessive hanging skin after bariatric surgery must be considered. In a survey of this influence, Ramalho et al. found that there was no significant correlation between sexual function (assessed by FSFI score) and excessive hanging skin or impairment due to excessive hanging skin, although there was an association with less concern about body image and fewer symptoms of depression [25].

In addition to sexual function, the research indicates differences in sexual behavior of obese girls and women. In a survey of obese female adolescents undergoing bariatric surgery, Zeller et al. found a greater increase in behaviors that pose a risk of sexually transmitted infections in those who underwent surgery. In addition, the proportion of pregnancies and live births as well as motherhood among teenagers (≤19 years) was higher in the group of young women who underwent bariatric surgery [26]. These results show the importance of considering sexual aspects in the postoperative period after bariatric surgery.

It is noticeable that all the presented studies focused on heterosexual women. Until now, there has been a lack of literature regarding non-heterosexual obese women undergoing bariatric surgery.

Regarding female sexuality and sexual function, the following key concepts can be formulated:

- There is strong evidence for improved sexual function as well as sexual quality of life and satisfaction with sexual life in obese women due to bariatric surgery.
- Although the prevalence of female sexual dysfunction decreases after bariatric surgery, it is still higher than in non-obese women.
- While there is an improvement of sexual function due to reduced weight, other aspects such as excessive hanging skin and social or mental aspects may be important for the sexual life of women after bariatric surgery.
- In the aftercare of bariatric surgery, changes in sexual behavior must be considered. There should be sufficient education of patients in terms of behaviors that pose risks for unintentional pregnancies and sexually transmitted infections.

### 3.2. Contraception

The effectiveness of contraceptive tools is a relevant aspect after bariatric surgery. Particularly directly after surgery, effective contraception is highly relevant since it is recommended to avoid pregnancy in the postoperative period (see Pregnancy subsection below). In this context, improved fertility after bariatric surgery must be considered, too (see Fertility subsection below). In the further follow-up period, women who have had bariatric surgery should be enabled to avoid unwanted pregnancy.

Oral contraceptives are among the most used contraceptives by women. These include combined oral contraceptives, colloquially called “the pill”, which contain the hormones estrogen and progestogen, as well as progestogen-only pills, colloquially called “the mini-pill” [27,28,29]. Regarding the effectiveness of oral contraception, the type of bariatric surgery is highly relevant. From a pharmacokinetic point of view, there might be a lack of effectiveness with procedures that include a malabsorptive component, such as Roux-en-Y gastric bypass or biliopancreatic diversion, due to potentially restricted bioavailability. Bioavailability could be affected by changes of gastric and intestinal solubility and pH as well as the loss of gastrointestinal transporters [30].

In contrast, recent research has found no significant difference in the concentration of esonogestrel plasma in women who used oral desogostrel before and after Roux-en-Y gastric bypass [31]. According to these results, oral contraceptives, including combined oral formulations, could be an effective tool after bariatric surgery. However, the number of cases in this initial study was very small, so further research is required to create enough scientific evidence on the effectiveness of oral contraceptives after bariatric surgery. Since there is a lack of such evidence, international guidelines recommend not using oral contraceptives due to possible decreased efficacy. Instead, several authors recommend implants for effective hormone-based contraception [30,32,33].

In terms of the effectiveness of oral contraception after sleeve gastrectomy, there has been a serious lack of scientific literature until now. The current scientific literature focuses primarily on the awareness of avoiding pregnancy in the postoperative period and the knowledge of using safe contraceptive methods and contraceptive counseling by attending physicians and the associated contraceptive behavior of women who have undergone bariatric surgery.

Regarding contraceptive behavior, Nimbi et al., found in a survey of 79 women who asked for bariatric surgery that more than a half did not use contraceptives, even though they were fertile and did not want to have a child. Women who used contraceptives had better FSFI scores and a better quality of life. The authors assumed that contraception may have a protective effect [17]. This conclusion has to be regarded critically, because covariate effects were not sufficiently discussed. In contrast, a British survey found that safe contraceptive methods were used by all women who participated in an anonymous and voluntary online questionnaire [34]. Regarding these results, the small number of cases (*n* = 42) as well as selection and ascertainment bias due to the study design must be kept in mind.

Clinicians and practitioners are often unaware of the importance of sufficient contraceptive counseling. An American survey showed that there was a lack of knowledge regarding the effectiveness of oral contraceptives after malabsorptive bariatric procedures among a large proportion of health care providers [35]. Another survey of stakeholders in bariatric and metabolic surgery in female minors found that after identifying and starting intervention by so-called key drivers, the proportion of use of safe contraceptive methods such as intrauterine devices increased [36]. These results underline the importance of being aware of effective contraception not only by patients but also by attending physicians.

Furthremore, they match the results of surveys in the Netherlands and the United States, among 230 and 35 women, respectively, undergoing bariatric surgery. Of these women, only 62.6% and 85.7%, respectively, were confirmed to have received contraceptive counseling. After surgery, 60% and 65.7% used safe contraception. In the Dutch survey, 16.1% used unsafe contraception and 23.9% used no contraception. In this study, there was a significant association between receiving contraceptive counseling and using safe contraceptive methods. In the USA too, the proportion of women who used contraception was higher in the group who received contraceptive counseling (95.7% vs. 66.7%) [32,37].

These results are consistent with those of Mengesha et al., who ascertained a lack of contraceptive counseling and no pregnancy counselling for about 25% and subjectively inadequate counseling for 66% of those who received any at all among women undergoing bariatric surgery. About a third of the women who underwent a procedure with malabsorptive aspects used oral contraceptives. In this survey, too, the postoperative use of contraceptives was independently associated with a discussion about contraceptives or pregnancy perioperatively [38].

Surveying clinical charts of more than 1000 female patients undergoing bariatric surgery, contraceptive counseling was documented for less than 30% [39], although a lack of documentation does not necessarily mean that no consultation took place. Nevertheless, nearly 1.6% of the women who underwent bariatric surgery became pregnant in the first 18 months postoperatively [39]. Women with a history of infertility especially have an increased probability of unprotected intercourse after bariatric surgery. The authors suggest that these women continue to have perceived infertility and that better counseling is needed [40]. The results of these surveys underscore the relevance of detailed consultation with treating physicians.

Regarding contraception, the following key concepts can be formulated:

- There is a lack of evidence regarding the effectiveness of oral contraceptives among women undergoing bariatric surgery.
- In order to avoid any risk that comes along with (unplanned) pregnancy, especially in the postoperative period, women who have bariatric procedures are discouraged from using oral contraceptives.
- In clinical practice, contraceptive and pregnancy counseling by bariatric physicians is often not performed at all or falls short.
- Contraceptive counseling can improve the use of safe contraceptive methods and therefore avoid risky pregnancies.
- Better awareness of or knowledge about contraceptive needs after bariatric surgery by physicians can improve the quality of contraceptive counseling.

### 3.3. Fertility

There is a complex association between infertility and obesity in women. For example, products of adipose tissue such as leptin, cytokines, and free fatty acids can affect the function of ovaries and endometrium. Peripheral insulin resistance as well as functional hyperandrogenism and hyperestrogenism may affect fertility, too [41]. While there is evidence for improved fertility due to weight loss, the role of bariatric surgery is a current research topic. As far as we know, there are no prospective randomized or epidemiologic surveys that can provide good evidence for improved fertility due to bariatric surgery. However, some pilot surveys and retrospective studies are beginning to draw conclusions.

In a large survey of 650 women, Menke et al. found an approximately 2.5-fold higher postoperative conception rate in nulliparous women with a preoperative history of infertility compared to those without. This increase includes the early postoperative timeframe (less than 18 months) when conception is contraindicated [40]. This research indicates that preoperative infertility may be improved as a result of bariatric surgery [42]. It underlines the importance of adequate contraception and contraceptive counseling (see Contraception subsection above).

In contrast, recent research shows a drop of anti-Mullerian hormone (AMH) levels, which could indicate impairment of the ovarian function after bariatric surgery. AMH is an important hormone in the cycle and conception rate of women. Produced in growing follicles of the ovaries, it correlates with ovarian function. There is a direct link between the AMH level and the number of maturable oocytes. Therefore, it is often used in fertility diagnostics. In women without a history of infertility, the AMH level is used to assess the ovarian reserve, or the number of functional oocytes; a high level can be associated with cycle irregularities. Therefore, AMH is increased in patients who suffer from polycystic ovary syndrome (PCOS) and is positively associated with serum levels of testosterone, androstendione, and dehydroepiandrosterone sulfate (DHEAS) [43,44,45,46]. In several pilot surveys, a decrease in AMH concentration after bariatric surgery independent of the surgical procedure was found. This drop persisted with the exclusion of PCOS patients [43,44,47,48]. The limitations of the current state of research as well as the complex interaction of fertility-promoting and fertility-inhibiting hormonal changes after bariatric surgery must be considered in this context, too.

Regarding obese women with a history of assisted reproductive technology, research indicates significant improvements in the outcomes of such technology as a result of bariatric surgery; while the total number of required gonadotropin units decreased after surgery, there was an increase in the number of follicles retrieved, top-quality metaphase II and fertilized oocytes and the number of top-quality embryos, the pregnancy rate, and the live birth rate [49].

Furthermore, results from the United Kingdom National Bariatric Surgery Registry, which represents a large epidemiologic survey show that bariatric surgery improves factors that underlie fertility and pregnancy outcomes, such as body weight, diabetes, menstrual dysfunction, and PCOS [50]. Moreover, a small survey in Iraq found improved fertility in women suffering from PCOS [51]. In the interpretation of these results, it must be considered that there are severe limitations to this survey due to the number of cases and the survey design.

As a consequence of the presented results, some authors recommend considering bariatric surgery as an alternative, or even a definitive, solution for women suffering from morbid obesity and infertility [52].

Regarding fertility, the following key concepts can be formulated:

- Obesity is linked to infertility by complex mechanisms and associations.
- There is evidence for significant improvement of various factors associated with infertility due to bariatric surgery.
- In the treatment of infertility in women, bariatric surgery is becoming an increasingly important issue.
- In order to improve the evidence, further prospective epidemiological research is required.

### 3.4. Pregnancy and Breastfeeding

Since many bariatric patients are female and of reproductive age, not only bariatric surgeons, but also obstetricians will have to deal with women undergoing bariatric surgery [53,54]. It is a well-known fact that obesity is associated with perinatal and maternal complications, e.g., fertility problems such as anovulation and miscarriage, metabolic disorders, congenital malformation, iatrogenic intervention, and perinatal morbidity and mortality [55,56,57,58].

Several studies show that bariatric surgery combined with lifestyle modifications leads not only to reduced cardiovascular morbidity, diabetes, cancer, and overall mortality but also to improved fertility (see Fertility subsection above). While pregnancy is a contraindication for bariatric surgery, research indicates improved pregnancy outcomes in the follow-up period and after the recommended latency period (see below) [50,59,60,61,62,63]. On the other hand, it has been proven that pregnant patients undergoing bariatric surgery are at higher risk for venous thromboembolisms, blood transfusion, induced labor, fetal growth restriction, and are at a significantly increased risk of needing further surgical procedures [59,63,64,65,66]. There is a higher risk of perinatal complications, and an operation-to-birth interval of less than two years can lead to more problems than longer intervals [67,68].

Prenatal diabetes control could be inaccurate, because dumping syndrome after bariatric surgery could falsify oral glucose tolerance tests, so some authors suggest monitoring pre- and post-meal blood glucose levels. Since there is a lack of research in terms of sufficient diagnostics for gestational diabetes in women with a history of bariatric surgery, researchers demand development of clinical guidelines [69,70].

Epidemiological research indicates an increased risk of intrauterine growth restriction in singleton pregnancies in obese women with a history of bariatric surgery [71]. These patients should be controlled regularly during their pregnancy and after birth by a team with experience in bariatric surgery and obstetrics and a nutritionist [54,67,68,72]. Some authors have even proposed that a midwife could coordinate the care of pregnant women who have undergone bariatric surgery [73].

In several studies and case reports, evidence could be found that after bariatric surgery women who become pregnant suffer from micronutrient deficiencies, e.g., vitamins A, D, C, B1, and B9 and selenium [74,75,76,77,78]. Furthermore, a study submitted that intake of proteins and omega 3 fatty acids could be difficult [77]. In terms of vitamin K, which is important in the prevention of intracranial bleeding in babies, there was no significant difference between women who underwent Roux-en-Y gastric bypass and those who did not. However, the authors remarked that the common practice of measuring vitamin K is unequal and there is insufficient evidence in bariatric patients [79]. Other authors found a higher level of anxiety in obese patients for whom pregnancy followed bariatric surgery, but they could not associate this with inadequate nutritional intake [80]. In addition, dermatological studies showed a higher risk of developing post-gestational dermatological manifestations such as phrynoderma or acquired acrodermatitis enteropathica [81].

This underlines the importance of screening for any deficiency and, especially for pregnant or breastfeeding patients, the long-term intake of nutritional supplements like vitamins, minerals, and trace elements [74,76,78,82,83]. By not doing this, severe complications for mother and child could arise [76]. In order to reduce fetal and maternal risk, the guidelines for postoperative management of bariatric patients of the American College of Obstetrical and Gynecology and the French National Authority for Health recommend contraception for at least 12–24 months [30]. In November 2019, Ciangura et al. presented clinical practice recommendations for pregnancy management following bariatric surgery [65].

Initial research indicates that breastfeeding after bariatric surgery has positive effects on the newborn. The composition of breast milk is sufficient concerning calories, macronutrients, and vitamin A [84]. Infants who were breastfed for at least six months showed lower fat mass and lower glucose levels, so the authors seconded the general recommendations of the WHO guidelines [78]. Breastfeeding could also be a protective factor regarding the development of obesity [85]. Breastfeeding is important for newborns and women should be encouraged to practice it.

A few years ago, there was a lack of structured studies, but with growing numbers of bariatric procedures, more and more studies were started, such as the multicenter prospective cohort Aurora study [61,86,87].

Regarding pregnancy and breastfeeding, the following key concepts can be formulated:

- Obesity-related pregnancy risk can decrease due to weight loss as a result of bariatric surgery.
- After bariatric surgery, there is an increased risk of micronutrient deficiencies in pregnant women. Sufficient supplementation and monitoring of pregnant women, especially after bariatric procedures that include malabsorptive aspects, is recommended.
- Due to the extreme weight loss, there is increased fetal risk in the postoperative period. Clinical guidelines recommend contraception/avoiding pregnancy for at least 12–24 months after surgery, depending on the source.
- There might be a lack of evidence for the validity of oral glucose tolerance testing to diagnose gestational diabetes in women with a history of bariatric surgery.
- Breastfeeding can be a protective factor in the development of obesity and is important for the newborn.
- There is no indication of problems with breast milk after bariatric surgery. Therefore, the WHO’s recommendations for breastfeeding also apply to women with a history of bariatric surgery.

### 3.5. Sexual Hormones and Polycystic Ovary Syndrome (PCOS)

Polycystic ovary syndrome (PCOS) is the most common hormonal disorder in reproductive-age women. PCOS affects the reproductive system by increased androgen production and disordered gonadotropin secretion. As a result, affected women can suffer from menstrual irregularity, hirsutism, and infertility. The metabolic features of PCOS are defects in insulin action and β-cell function, which lead to an increased risk of glucose intolerance and type 2 diabetes. There is a mutual association between obesity and PCOS, and the prevalence of PCOS is significantly increased in obese women; while it is about 7% in the total female population, it occurs in 40%–80% of obese women, depending on the source [88]. In addition, menstrual disorders that might be associated with PCOS, such as dysmenorrhea, heavy menstrual bleeding, or irregular bleeding, are more common in obese women [34].

A retrospective survey of 44 women with PCOS who underwent bariatric surgery showed a significant reduction of androgen levels and the proportion of women meeting the criteria for hyperandrogenism and irregular menses. In addition, it could be shown that the ovarian volume was independently associated with changes in hemoglobin A1C (HbA1C) and triglycerides. In turn, both HbA1C and triglycerides decreased significantly after bariatric surgery. Preoperative free testosterone was linked with changes in total cholesterol and non-high-density lipoprotein cholesterol (non-HDL-C). In a subsequent survey, the authors found a significant reduction of ovarian volume in severely obese women suffering from PCOS after bariatric surgery. The authors concluded that bariatric surgery improves key diagnostic features in women with PCOS and hyperandrogenism [89,90]. Another survey with a follow-up of four years confirmed the persistence of these hormonal changes. An exception was estradiol, which did not differ significantly four years after bariatric surgery [22]. Additionally, research has shown normalization of menstrual irregularities in PCOS patients after bariatric surgery [47].

Results of a large meta-analysis showed a prevalence of PCOS in 36% of severely obese women. There was resolution for 96% of those women after bariatric surgery. In women, total testosterone, which is associated with hirsutism and menstrual dysfunction, decreased after bariatric surgery. In addition, sex-hormone binding globulin (SHBG) increased while serum estradiol decreased in women after bariatric surgery. SHBG, a protein synthesized and secreted by the liver, is responsible for the transport of androgens and estrogens. Its synthesis depends on the balance between stimuli such as estrogens and thyroid hormones, and inhibitory influences such as androgens and insulin, liver fat, and inflammatory mediators. The fact that the SHBG levels increased while estradiol decreased after bariatric surgery led the authors to conclude that inhibitory influences arising from adipose tissue dominate the picture in severe obesity [91].

These results underline the complex mechanisms on which obesity-associated gonadal dysfunctions are based. Based on good cure rates, authors recommend offering bariatric surgery to severely obese patients who suffer from obesity-associated gonadal dysfunction [42,91].

Another important hormone in the metabolism of adipose tissue is adiponectin. Decreased levels of this fat-derived hormone are supposed to be associated with insulin sensitivity, and with glucose and lipid metabolism and cardiovascular disorders. On the other hand, the production of adiponectin is affected by obesity and associated pathologies [92]. After bariatric surgery, there is an increase in adiponectin levels, and the increase is higher in women without PCOS than in those with PCOS [93].

The nonspecific inflammatory marker C-reactive protein (CRP) is more often increased in people who are obese and/or have a metabolic disorder. In turn, increased CRP is associated with a higher risk of coronary heart disease in metabolically preloaded people [94]. Recent research has shown that weight loss as a result of bariatric surgery is an effective method to decrease obesity-associated CRP levels [93].

Regarding sexual hormones and polycystic ovary syndrome (PCOS), the following key concepts can be formulated:

- PCOS and other complex dysregulations of the female sex hormone balance are associated with obesity.
- Weight loss due to bariatric surgery improves PCOS significantly and can regulate hormonal disorders such as obesity-related gonadal disorders.
- In the treatment of obesity-related gonadal disorders/PCOS in severely obese women, bariatric surgery should be taken into consideration as a therapeutic option.
- Regarding the nonspecific inflammatory marker CRP and the metabolic protecting hormone adiponectin, first scientific results indicate positive effects due to bariatric surgery.

### 3.6. Menopause and Osteoporosis

Large epidemiologic surveys show an increased risk of osteoporosis in women. While the prevalence increases in both sexes with age, it reaches a climax of close to 80% in women up to 80 years. In addition to vitamin D deficiency, hormonal changes after menopause, such as decreased levels of estrogen, are responsible for the increased risk. In terms of bone mass and bone stability, the complex effects and interactions of estrogen are particularly important. Hormonal changes during the menopausal transition period are also associated with typical bothersome symptoms such as hot flashes, night sweats, vaginal atrophy and dryness, dyspareunia, sleep disturbance, and mood swings, which occur to varying degrees in a majority of women [95,96].

Recent research indicates a decrease in bone mineral density and bone mineral content (by DEXA scan) and therefore deterioration of bone quality after bariatric surgery [97]. Researchers suggest that bariatric surgery may result in bone loss due to potential vitamin D deficiency, mechanical unloading from weight loss, and changed hormonal secretion such as reduced leptin and estrogen in perimenopausal women [98].

While there is more evidence for increased risk of osteoporosis after Roux-en-Y gastric bypass than after sleeve gastrectomy, initial research indicates that there is no significant difference [97]. Roux-en-Y gastric bypass is suggested to be involved in impaired absorption of calcium and vitamin D. As a result, secondary hyperparathyroidism accompanied by increased degradation of bone material may occur [99]. The greatest amount of bone loss takes place within the axial skeleton, such as spine and ribs, an area that is particularly vulnerable even before bariatric surgery [97].

Regarding the complex hormonal changes associated with both severe obesity and bariatric surgery, the question of the role of bariatric surgery in menopausal symptoms arises. First research has shown that bothersome menopausal symptoms such as hot flushes become rarer after bariatric surgery [100].

Comparing women who did not undergo Roux-en-Y gastric bypass with a group of women matched by age, sex, and weight who did, the latter had better blood values regarding total cholesterol, low-density lipoprotein, and triglycerides but a higher mean parathyroid hormone level. These results matched with the observation of vitamin D deficiency in the later postoperative period in a prospective five-year follow-up survey [99].

Although there is hypercaloric energy intake by most severely obese women, recent research has shown that this population often presents with micronutrient deficiencies. A Chilean survey of 66 women prior to bariatric surgery found a prevalence of more than 70% of vitamin D deficiency and 66% of increased parathyroid hormone levels [101].

Regarding osteoporosis and menopausal symptoms, the following key concepts can be formulated:

- Although there is increased intake of macronutrients, severely obese women often suffer from micronutrient deficiencies, especially vitamin D. Even before bariatric surgery, severely obese women represent a vulnerable group in terms of the development of osteoporosis.
- Due to continued diminished intake and affected absorption, bariatric surgery can increase that risk.
- Prophylactic substitution of calcium is recommended; vitamin D and parathyroid hormones should be observed closely in the postoperative period.
- There is a lack of evidence regarding the influence of bariatric surgery on menopausal symptoms. Initial research indicates a positive effect.

### 3.7. Pelvic Floor Disorders and Urinary Incontinence

Pelvic floor disorders (PFDs) is a collective term for a broad spectrum of clinical conditions. They mainly include urinary incontinence, fecal incontinence, pelvic organ prolapse, and sexual dysfunction. The prevalence increases with age, and it is estimated to be between 2% and 42% in adult females [102]. Obesity is known as an important risk factor for developing pelvic floor disorders. The greater intra-abdominal pressure, weakened pelvic floor muscles, and structural damage or neurologic dysfunction contributing to prolapse and incontinence are possible mechanisms for the effects of obesity on PFDs.

Al-Shaikh et al. investigated the frequency of PFDs and their effects on quality of life in a group of obese women (*n* = 75) versus a control group of non-obese women (*n* = 91). They found increased frequency of pelvic organ prolapse as well as urinary and fecal incontinence in the group of obese women, 63% versus 33% in the non-obese control group [103]. Symptoms of PFDs include pelvic pain, pressure, urinary and anal incontinence, incomplete emptying of bladder and bowel, dyspareunia, and pelvic organ protrusion through the vagina, resulting in reduced quality of life and significant medical, social, and emotional distress for many women [104].

The 20-item Pelvic Floor Disability Index (PFDI-20) is a validated questionnaire for evaluating pelvic floor disorders in women, consisting of 20 questions that explore the symptoms of genital prolapse, colorectal-anal symptoms, and urinary symptoms [105]. Recent studies focus mainly on the symptom complex of urinary incontinence. Possible reasons may be the high prevalence of urinary incontinence in contrast to prolapse symptoms and fecal incontinence. Another reason could be that pelvic organ protrusion symptoms are less known and obese women seem to be less knowledgeable compared to the general population [106]. A prospective study in Turkey interviewed 53 women undergoing bariatric surgery and assumed a connection between decreased symptoms of urinary incontinence and increased quality of life [107]. The success of bariatric surgery in terms of weight loss is well documented within the literature. Its effects on PFDs in obese women are now under investigation.

Data obtained in recent studies show improvement in PFDs and especially urinary incontinence after bariatric surgery. In two independent studies (*n* = 240 and *n* = 366), a statistically significant reduction in ICIQ-UI score was found; 33–44% of women reported a complete cure of urinary incontinence after bariatric surgery [105,108,109,110].

To assess the symptoms and impacts of urinary incontinence, the International Consultation on Incontinence Questionnaire-Urinary Incontinence (ICIQ-UI) is the most commonly used instrument. It is a validated questionnaire for initial diagnosis, management, and patient follow-up. It allows subdivision into classes of symptom severity (slight: 1–5; moderate: 6–12; severe: 13–18; very severe: 19–21) [110].

A prospective cohort study in Israel investigated the effect of significant weight loss after laparoscopic sleeve gastrectomy or gastric bypass on lower urinary tract symptoms and pelvic floor disorders in 150 women. The most frequent preoperative symptom was urinary incontinence, found in more than one-third of the women. The mean BMI before and 3–6 months after surgery were 42 and 32 kg/m^2^, respectively. Preoperatively, 37% of the women suffered from urinary incontinence. Postoperatively, 88% of those women reported improvement and 48% reported complete resolution of their urinary incontinence following weight loss. In addition, the authors found significant improvement in pelvic organ prolapse symptoms and colorectal-anal symptoms. This is also supported by a systematic review and meta-analysis from 2018 [111]. However, it remains unclear whether an improvement in the score also means an improvement in the clinical symptoms in everyday life [112,113].

The follow-up periods of the presented studies were short; the longest was a survey with an observation period of five years. In this retrospective study, the survey respondents were divided into four groups based on the quartile of weight loss. An inverse relationship between the amount of weight loss and subsequent pelvic organ prolapse and urinary incontinence symptoms was found [114].

O’Boyle et al. investigated the effect of weight loss depending on subtypes of urinary incontinence. They subdivided it into stress urinary incontinence, mixed urinary incontinence, and overactive bladder. In two studies they found mixed urinary incontinence was the most prevalent subtype, followed by stress urinary incontinence and overactive bladder. Cure rates were controversial. There was still no evidence to show which subtype is treated best with weight loss [109,110]. Although the improvement of PFD and urinary incontinence through weight loss is clear, the effect of lifestyle changes after bariatric surgery must also be considered. Factors that can be associated with improved urinary incontinence include postoperative fluid restriction and increased physical activity through weight loss.

Regarding pelvic floor disorders and urinary incontinence, the following key concepts can be formulated:

- Pelvic floor disorders, and particularly urinary incontinence, are associated with obesity.
- Pelvic floor disorders and urinary incontinence can be improved by weight loss after bariatric surgery.
- There is a lack of research regarding the effects of bariatric surgery on prolapse symptoms and anal incontinence.
- In order to improve the evidence, further prospective epidemiological research with longer follow-up periods and more cases is required.

### 3.8. Female-Specific Cancer

Overweight and obesity are associated with an increased risk of a number of cancer diseases. Among women, sex-specific cancer represents a large proportion of malignant diseases: breast cancer is the most common and cervical cancer is the sixth most common cancer entity globally [115]. Decreased estrogen levels have been supposed to increase the risk of developing breast and endometrial cancer and are therefore among the reasons for the increased incidence of malignant diseases in women [116]. Endometrial hyperplasia is a precancerous condition that arises in the presence of chronic exposure to estrogen unopposed by progesterone, such as in PCOS and obesity [42]. Therefore, regarding sex-specific aspects in the bariatric treatment of women, the question of the influence of surgery-induced weight loss on sex-specific cancer arises.

A histologic study of the endometria of morbidly obese women who presented for bariatric surgery found a prevalence of endometrial hyperplasia of about 10%. Bariatric surgery not only changed the risk variables for the development of endometrial cancer but also improved the quality of physical health significantly [117]. Therefore, it can be assumed that severely obese women represent a risk group in terms of endometrial cancer. Although a positive effect of weight loss on endometrial dysplasia (risk) appears to be plausible, there is a lack of research regarding the influence of bariatric surgery until now.

While there were no prospective randomized research results available at the time of this review, researchers recently published a case of a female adolescent with brad-1 endometrial adenocarcinoma, PCOS, and type 2 diabetes. In order to provide fertility-preserving treatment, local hormonal therapy was given, but it failed. After bariatric surgery and normalization of body weight, the patient showed complete response to local treatment of the endometrial cancer [118].

As promising as these observations appear to be, further research is needed on the effects of bariatric treatment on reducing risks and providing treatment approaches for female-specific cancers, as well as the underlying mechanisms. Reviewing the current status of research, researchers conclude that bariatric surgery has a positive effect on endometrial hyperplasia, making surgically induced weight loss a potentially attractive option for endometrial cancer prevention and treatment [42,119]. A systematic review and meta-analysis including close to 900,000 patients found a reduced risk of endometrial cancer postoperatively in those receiving bariatric surgery [120].

A Cochrane review was aimed at determining the impact of behavioral and lifestyle weight-loss interventions of endometrial cancer. Three randomized controlled trials were included in this standardized review paper. In contrast to the results of bariatric surgery, behavior and lifestyle interventions were not associated with improved overall survival, nor cancer-specific survival or improved health-related quality of life. Nevertheless, the authors pointed out that there was a lack of evidence in this research field [121].

In a Swedish long-term follow-up survey of about 18 years on average, a reduced incidence of overall cancer and female-specific cancer and a reduced risk of endometrial cancer in obese women who underwent bariatric surgery were found compared to a matched group of women who received conservative weight-loss treatment. In addition, the researchers found a link with serum insulin: women with pre-existing medium or high insulin levels had higher risk reduction of female-specific cancer due to bariatric surgery [122]. Research indicates that bariatric surgery has a positive effect on endometrial hyperplasia, making surgically-induced weight loss a potentially attractive option for endometrial cancer prevention and treatment. [42]

As a result of these promising treatment results, some authors demanded that strong consideration be given to surgical weight loss as a therapeutic option in suitable endometrial cancer candidates by a gynecologist [123]. Moreover, from an economic point of view, bariatric surgery appears to be a cost-effective intervention, at least for improving quality of life and losing weight, in women with low risk, early stage endometrial cancer [124]. Identifying surgery-specific aspects is difficult because non-surgery weight loss attempts usually do not result in sufficient weight loss, at least on a long-term basis [125].

Possibly as a result of the promising research outcomes of bariatric surgery in the treatment and prevention of endometrial cancer, the current Cochrane protocol “Fertility-sparing treatment for atypical endometrial hyperplasia and endometrial cancer” includes bariatric surgery as a treatment option [126]. The results of this systematic analysis remain to be seen.

In a review that included four scientific studies (prospective as well as retrospective), Hoel et al. concluded that the risk of endometrial and breast cancer decreased due to bariatric surgery in women, although details on the underlying mechanisms and cancer entities remain unclear [127]. With regard to breast cancer, there is even less scientific evidence regarding the influence of bariatric surgery. A retrospective cohort study of BMI- and aged-matched women with and without bariatric surgery showed that the surgery was associated with reduced risk of both pre- and postmenopausal breast cancer [116].

Facing a highly relevant public health topic, this survey was widely discussed in the scientific community. Researchers remarked that there were several limitations in this survey, including that it disregarded further risk factors for developing breast cancer and protective factors and did not analyze stratified BMI or the timing of cancer occurrence and bariatric procedures in detail [128]. According to the authors, not all of this information was available [129]. However, the well-developed design and large number of cases allow the survey to offer important findings about potential approaches to reducing the risk of breast cancer.

Regarding female-specific cancer, the following key concepts can be formulated:

- Obesity is associated with increased risk of female-specific cancer, such as breast and endometrial cancer.
- Weight loss as a result of bariatric surgery decreases the incidence of endometrial and breast cancer in severely obese women.
- First research indicates that bariatric surgery can lead to regression and healing of endometrial hyperplasia, a precancerous condition that can develop into endometrial cancer.

### 3.9. Metabolism, Outcome, and Quality of Life

Although important differences between the male and female metabolism are well known, there is still a lack of research regarding the sex-specific influence of bariatric surgery on metabolic aspects. First research from rodent models showed important differences in hepatic lipid metabolism after sleeve gastrectomy; while male rodents had a significant reduction in hepatic lipids, there was no reduction in female rodents [130]. However, similar results from human surveys are lacking. In terms of sex-specific differences in the outcome of bariatric surgery, there are controversial results. An English survey analyzed the outcome of bariatric surgery in men and women. After matching the patients for age, BMI, diabetes, insulin, and continuous positive airway pressure (CPAP) treatment, there were no significant differences in outcome regarding weight loss and improvement of obesity-related comorbidities [131].

Other results were in the US: In a multicenter analysis of more than 60,000 patients, male patients lost less weight, and had a smaller decrease in comorbidities and more complications. These results correspond with those of the German Bariatric Surgery Register, which found significantly higher BMI, age, and incidence of comorbidities among men who underwent bariatric surgery [132,133]. Nevertheless, the results of the US survey showed that men were significantly more satisfied with their operation. Regarding the sample, male patients were older on average, and had a higher BMI and more comorbidities than female patients. They represented only 22% of the whole, which is congruent with the nationwide data. The authors suspected that men tend to seek treatment later [132]. This fits with the results of Wee et al., who found differences concerning self-evaluated quality of life, social stigma, and the utility of bariatric surgery in achieving personal goals depending on sex as well as ethnicity [134].

These conclusions match those of Young, Phelan, and Nguyen, who suspected that there were barriers to access of bariatric treatment for men. In their decade (2002–2011) analyzing about 811,000 patients, the number of male patients undergoing bariatric surgery was much smaller than the number of female patients. In addition, male patients tended to have higher severity of illness, with higher risk-adjusted serious morbidity and mortality rates [135]. Commenting on their research, researchers from Iran also reported that the proportion of women among bariatric patients was significantly higher [111].

The presented research indicates that differences in outcome after bariatric surgery between men and women result from differing prevalence rather than effects of the surgery. However, due to the small number of cases and the retrospective survey design, further research is required.

There is evidence for improved quality of life after bariatric surgery in both men and women [136]. Unfortunately, only a small proportion of studies address sex-specific aspects or compare outcomes between men and women in this field. Further scientific investigation focusing on sex-specific aspects in the outcome of bariatric surgery is needed.

Regarding metabolism, outcome, and quality of life, the following key concepts can be formulated:

- Although the prevalence of obesity is equal in men and women or even higher in men, men represent a minority among patients who undergo bariatric surgery.
- If treated, male patients have a higher BMI and comorbid risk profile on average. As a result, the benefit of the bariatric procedure is as high as it is in women. Nevertheless, satisfaction with the operation is greater in men.
- Further research about sex-specific aspects in the outcome of bariatric surgery, especially regarding metabolic aspects, is needed.

### 3.10. Other

Several surveys found that sex-specific special features after bariatric surgery are highly relevant, but they do not offer a sufficient basis of research to present and discuss them in a separate category. Surveying abdominoplasty requests after bariatric surgery, Ahmed et al. found some differences between men and women: for female patients the decisive reason for seeking abdominoplasty after bariatric surgery was clothing/fashion, and for men it was dermatological problems. In addition, women sought abdominoplasty sooner after the operation and at a younger age [137]. However, another survey found no differences in self-image or quality of life due to excessive skin after bariatric surgery. Overall, patients who had less excessive skin had a better self-image and quality of life [138].

There is good evidence for improvement of obstructive sleep apnea as a result of bariatric surgery due to its strong association with overweight and obesity. Initial research indicates an even greater benefit for improved obstructive sleep apnea and associated symptoms in women than men [139]. However, further research on this topic is needed in order to generate sufficient evidence of sex-specific aspects in the relationship of obstructive sleep apnea and bariatric surgery.

Regarding other factors, the following key concepts can be formulated:

- Similar to the decision for bariatric surgery, sex-specific aspects might affect the decision to undergo plastic surgery such as abdominoplasty after weight loss due to bariatric surgery.
- Abdominoplasty to reduce excessive hanging skin after bariatric surgery can improve quality of life similarly in men and women.

## 4. Discussion

### 4.1. Summary

This review was aimed at working out sex-specific aspects in the bariatric treatment of severely obese women. After an overview of the current status of research, clinically relevant aspects and recommendations were presented. Key aspects were given for each category as a summary.

Based on a systematic literature search, the categories sexuality and sexual function; contraception; fertility; sex hormones and polycystic ovary syndrome; menopause and osteoporosis; pregnancy and breastfeeding; pelvic floor disorders and urinary incontinence; female-specific cancer; and metabolism, outcome, and quality of life were identified as sex-specific. Further research articles that met the inclusion criteria but could not be assigned to any of those categories were discussed in the “other” category.

Further categories that may be sex-specific but do not discuss sex-specific special features of women or relevant aspects concerning treatment are social and mental aspects and eating behavior and eating disorders. These categories are not included in this review paper.

The presented research clearly shows improvements in a number of female-specific disorders: there is strong evidence for improvements in sexual disorders, pelvic floor disorders, infertility, and risk of endometrial and breast cancer in severely obese women as a result of bariatric surgery. On the other hand, an increased risk of osteoporosis and unplanned and risky pregnancy is noticeable. These aspects require special attention, such as close observation and sufficient counseling. In addition, several aspects were presumed to be sex-specific, but these aspects and the resulting consequences for clinical practice were not sufficiently discussed. Therefore, until now there has been a lack of evidence concerning sex-specific aspects in several fields associated with bariatric treatment, such as sex-specific metabolism and outcomes after bariatric surgery.

### 4.2. Limitations

This review paper represents the current scientific status of sex-specific aspects in the bariatric treatment of severely obese women. It presents the current state of research as well as current approaches and areas of focus that were worked out systematically by the literature search. As a result, a lack or an underrepresentation of research, as was found in some topics, may also persist within this review. The same is possible for overrepresentation of research fields. Additionally, the reduction of listed search terms when performing the literature search represents a limitation due to the possible presence of articles that do not include these terms but include relevant aspects.

Next, this paper reproduced the results of research that has already been conducted. The literature was read carefully and publications that did not meet the criteria of good scientific practice and systematic approaches were excluded. Nevertheless, the authors cannot guarantee the adequacy and truthfulness of each of the presented surveys.

This manuscript does not include a quantitative analysis. Therefore, an assessment of risk of bias, as PRISMA-P criteria recommend for data synthesis and meta-analysis, was not performed for the included surveys. Some of the surveys show severe limitations, such as a very small number of cases or selection bias. As a result, the limitations of this review paper follow those of the presented surveys. In order to consider the limitations by the interpretation, they were named and discussed critically after the presentation of the respective results.

### 4.3. Further Approaches

Several sex-specific categories in the bariatric treatment of severely obese women were worked out. Although these aspects are particularly relevant, they are not sufficiently addressed in clinical practice. While many scientific publications focus on the bariatric treatment of severely obese women, only a small proportion of studies discuss sex-specific aspects of their selected population. Since there is good evidence for the relevance of these aspects, their consideration is desirable in further approaches.

Next, there are several sex-specific special features not only in treatment but also in anatomy, metabolism, and physiology, which may result in different outcomes of bariatric surgery. Although highly relevant, there is a lack of scientific evidence for this aspect of treatment. Further scientific approaches that can provide good evidence, such as large epidemiologic surveys or prospective randomized controlled trials, are needed. Therefore, we recommend further quantitative synthesis regarding the individual categories based on this qualitative review. An improvement in the current status of research would empower physicians and developers of clinical guidelines to take sex-specific differences more into consideration.

### 4.4. Valorization

This paper aimed to point out female-specific aspects in the bariatric treatment of severely obese women. Especially pregnancy, family planning, and sex-specific comorbid hormonal and cancer risks and diseases have to be considered.

Regardless of the strong evidence presented in this manuscript, the majority of clinical guidelines have not sufficiently considered these female-specific aspects in terms of either indications for bariatric surgery or the choice of procedure [9,11].

In this context it must be kept in mind that the guidelines are comparatively new and, therefore, undeveloped. For example, the first German S3 guidelines were published in 2014. The revised guidelines of 2018 take into account comorbid diabetes mellitus, especially with regard to the increasing evidence for metabolic surgery. However, sex-specific aspects are not mentioned with regard to the selection of surgical procedure. For example, planned pregnancies are not listened in this context, although there is strong evidence for differences regarding post-bariatric surgery risks during pregnancy depending on the procedure.

This paper points out the current status of research regarding female-specific aspects of bariatric treatment. As a result, relevant aspects in clinical practice are worked out. A general overview providing the most important clinical aspects is additionally presented in Figure S1. Next, this manuscript aims to contribute a basis for further development of guidelines. The results of this literature review may provide a basis for including sex-specific aspects in revised versions of clinical guidelines.

## 5. Conclusions

This paper aimed to point out sex-specific special features that are important in the bariatric treatment of severely obese women. By a systematic literature search on the topics of sexuality and sexual function; contraception; fertility; sex hormones and polycystic ovary syndrome; menopause and osteoporosis; pregnancy and breastfeeding; pelvic floor disorders and urinary incontinence; female-specific cancer; and metabolism, outcome and quality of life were elucidated. Each is illuminated and discussed separately, with a focus on clinically relevant aspects.

There is evidence for the improvement of a number of diseases and disorders in severely obese women due to bariatric surgery. However, sex-specific risks after undergoing bariatric procedures must be considered in the aftercare. Currently, the recommendations regarding bariatric procedures in the guidelines on the treatment of obesity focus on reducing weight and, therefore, metabolic risks. This paper shows that bariatric procedures offer potential in the treatment of a number of disorders and diseases that goes far beyond these goals. Especially regarding hormone-related disorders, bariatric surgery offers promising results. In this context, the current clinical practice of using indications for surgical treatment based on body weight and metabolic aspects has to be questioned. Further research and stronger consideration of bariatrics in the gynecologic treatment of obese and severely obese women are desirable.

## Figures and Tables

**Table 1:** Databases and search engines used in this research.

Search Engine	Databases
EBSCO Publishing	Academic Search Premier American Psychological Association (APA)/PsycINFO Open Dissertations
Cochrane Library	Cochrane
PubMed	HealthSTAR Medline
Scopus	Scopus (Elsevier)

**Table 2:** Literature search tracking sheet.

Dates of Search	Database	Publication Years	Further Search Settings	Search Terms	Hits
30/09/2019–05/10/2019	MEDLINE	Last 5 years	In title	bariatric AND women	113
				bariatric AND female	21
				bariatric AND sex	11
				bariatric AND gender	9
07/10/2019	Academic Search Premier	Since 10/2014	In title/subjects	bariatric AND women OR female OR woman OR females	66
				bariatric AND sex OR gender	10
07/10/2019	PsycInfo	Since 10/2014	In title/subjects	bariatric AND women OR female OR woman OR females	18
				bariatric AND sex OR gender	2
08/10/2019	Cochrane	–	In title/subjects	bariatric	13
08/10/2019	Scopus	Since 2015	In title	bariatric AND women OR female OR sex OR gender	136
Sum					399

## Supplementary Materials

The following are available online at https://www.mdpi.com/1660-4601/17/8/2734/s1, Figure S1: General overview about the most important clinical aspects in the bariatric treatment of severely obese women, Table S1: Complete results of systematic literature search, Table S2: Screened records of systematic literature search and assignment into categories, Table S3: Detailed information regarding the included literature, Table S4: Criteria for Literature search and inclusion.

## Abbreviations

AMH	anti-Mullerian hormone
BMI	body mass index (kg/m^2^)
CRP	C-reactive protein
DHEAS	DHEAS
FSFI	Female Sexual Function Index
HbA1C	hemoglobin A1C
ICIQ-IU	Incontinence Questionnaire-Urinary Incontinence
non-HDL-C	non-high-density lipoprotein cholesterol
PFDI	Pelvic Floor Disability Index
SD	standard deviation
WHO	World Health Organization
