# Supplementary material for: Review: Sex-Specific Aspects in the Bariatric Treatment of Severely Obese Women
Source: Int J Environ Res Public Health. 2020 Apr 15;17(8):2734. doi: 10.3390/ijerph17082734 (PMC7216185; doi:10.3390/ijerph17082734)
Supplement: Supplementary file 1 [file ijerph-17-02734-s001.zip › ijerph-736605 -Figure S1.pdf]

- There is strong evidence for improved sexual function as well as sexual quality of life and satisfaction with sexual life in obese women due to bariatric surgery.
- Obesity is linked to infertility by complex mechanisms and associations which can be improved as a result of bariatric surgery
- In the aftercare of bariatric surgery, changes in sexual behavior must be considered. There should be sufficient education of patients in terms of behaviors that pose risks for unintentional pregnancies and sexually transmitted infections.
- In order to avoid any risk that comes along with (unplanned) pregnancy, especially in the postoperative period, women who have bariatric procedures are discouraged from using oral contraceptives.
- After bariatric surgery, there is an increased risk of micronutrient deficiencies in pregnant women. Sufficient supplementation and monitoring of pregnant women, especially after bariatric procedures that include malabsorptive aspects, is recommended.
- Due to the extreme weight loss, there is increased fetal risk in the postoperative period. Clinical guidelines recommend contraception/avoiding pregnancy for at least 12–24 months after surgery, depending on the source.
- There is no indication of problems with breast milk after bariatric surgery. Therefore, the WHO's recommendations for breastfeeding also apply to women with a history of bariatric surgery.
- Weight loss due to bariatric surgery improves PCOS significantly and can regulate hormonal disorders such as obesity-related gonadal disorders.
- Regarding the nonspecific inflammatory marker CRP and the metabolic protecting hormone adiponectin, first scientific results indicate positive effects due to bariatric surgery.
- Although there is increased intake of macronutrients, severely obese women often suffer from micronutrient deficiencies, especially vitamin D. Due to continued diminished intake and affected absorption, bariatric surgery can increase that risk.
- Pelvic floor disorders and urinary incontinence can be improved by weight loss after bariatric surgery.
- Weight loss as a result of bariatric surgery decreases the incidence of endometrial and breast cancer in severely obese women. First research even indicates that bariatric surgery can lead to regression and healing of endometrial hyperplasia.
- Although the prevalence of obesity is equal in men and women or even higher in men, men represent a minority among patients who undergo bariatric surgery. If treated, male patients have a higher BMI and comorbid risk profile on average.

Figure S1. General overview about the most important clinical aspects in the bariatric treatment of severely obese women.
