# Supplementary material for: Review: Sex-Specific Aspects in the Bariatric Treatment of Severely Obese Women
Source: Int J Environ Res Public Health. 2020 Apr 15;17(8):2734. doi: 10.3390/ijerph17082734 (PMC7216185; doi:10.3390/ijerph17082734)
Supplement: Supplementary file 1 [file ijerph-17-02734-s001.zip › ijerph-736605-TableS1-S4.pdf]

**Supplementary Materials**

**Table S1: complete results of systematic literature search**

| All results | Note | Reason for exclusion | Category |
|---|---|---|---|
| **MEDLINE Bariatric AND Women** | | | |
| (Ganesan & Choy, 2019) | Only comment / no research article | | (Breast cancer) |
| (Feigelson et al., 2019a) | Only response to comment / no research article | | (Breast cancer) |
| (Maslin, James, Brown, Bogaerts, & Shawe, 2019) | | - | Pregnancy |
| (Koehler et al., 2019) | | No sex-specific aspects | (Phanse angle/ predictor) |
| (Keller et al., 2019b) | | | Quality of live<br>Urinary incontinence |
| (Shah et al., 2019) | | | Contraception |
| (Damhof et al., 2019) | | | Contraception |
| (Bartholomay, Berlin, McInerney, & Garcia, 2019) | | | Pregnancy /vitamins |
| (Luhrs et al., 2019) | | | Osteoporosis |
| (Sande-Lee et al., 2019) | | No sex-specific aspects | (Else: hypothalamic gliosis) |
| (Dereppe, Forton, Pauwen, & Faoro, 2019) | | No sex-specific aspects | (Lungs and breathing) |
| (Nogué et al., 2019) | | No sex-specific aspects | (Eating behavior/ disorders) |
| (Balestrin, Urbanetz, Barbieri, Paes, & Fujie, 2019) | | | pregnancy |
| (Pichlerova et al., 2019) | | | Sexuality/sexual function |
| (Feigelson et al., 2019b) | | | Breast cancer |
| (Reichmann, Todeschini, Setter, Vilela, & Radominski, 2019) | | No sex-specific aspects | (Eating behavior/ disorders) |
| (Menke et al., 2019) | | | Infertility |
| (Hubert et al., 2019) | | No sex-specific aspects | (Eating behavior/ disorders) |
| (Steffen et al., 2019) | | | Sexuality/sexual function |
| (Cherick et al., 2019) | No sex-specific aspects? | | Quality of life & Self-esteemed health |
| (Lechmiannandan et al., 2019) | | | Sexuality/sexual function |
| (Paka et al., 2019) | | | Urinary incontinence |
| (Vieira et al., 2019) | | No sex-specific aspects | (Eating behavior/ disorders) |

| | | | |
|---|---|---|---|
| (Steffen et al., 2019) | | Duplicate | |
| (Beato, Ravelli, Crisp, & de Oliveira, 2019b) | | Only correction of the author’s spelling in the original article & no sex-specific aspects | (Phase angle/ bio impedance) |
| (Beato, Ravelli, Crisp, & de Oliveira, 2019a) | | No sex-specific aspects | (Phase angle/ bio impedance) |
| (Garretto et al., 2019) | | | Pregnancy/ breastfeeding |
| (Faria, Faria, de Gouvêa, & Amato, 2019) | | | Pregnancy/ adherence |
| (Zhang, Gao, Liu, & Liu, 2018) | | | Urinary incontinence |
| (Abiad et al., 2018) | | | Sexual hormones/ PCOS |
| (Harreiter et al., 2018) | | | Pregnancy |
| (Graham et al., 2018) | | | Contraception |
| (J. M. Gómez-Martin et al., 2018) | | No sex-specific aspects | (Nutrition/ vitamins) |
| (Pinhel et al., 2018) | | No sex-specific aspects | (Epigenetic aspects) |
| (Vincentelli et al., 2018) | | | Fertility |
| (Palma et al., 2018) | | No sex-specific aspects | (Antibiotic prophylaxes) |
| (B. M. Mengesha et al., 2018) | | | Contraception |
| (Ficaro, 2018) | No sex-specific aspects? | | Social aspects |
| (de Campos et al., 2018) | | No sex-specific aspects | (Lungs and breathing) |
| (J. P. Christ & Falcone, 2018) | | | Sex hormones/PCOS |
| (Gabriel, Tavakkoli, & Minassian, 2018b) | | | Urinary incontinence |
| (Wein Alan J., 2018) | Only comment, no research article | No additional scientific value | Urinary incontinence |
| (TERRA et al., 2017) | | No sex-specific aspects | (Physical activity) |
| (Jans, Matthys, et al., 2018) | | | Pregnancy / (mental disorders) |
| (Sarwer et al., 2018) | | | Sex hormones / Quality of life Sexuality/ Sexual function |
| (K. L. McLean, Moore, Miketinas, & Champagne, 2018) | | No sex-specific aspects | (Eating behavior/ disorders/ethnic aspects) |
| (Hazart et al., 2017) | | | Pregnancy / Childbearing |
| (Gimenes et al., 2018) | | | Pregnancy / Childbearing/ Breastfeeding |

| | | | |
|---|---|---|---|
| (Schnor et al., 2017) | | No sex-specific aspects | (Epigenetic aspects) |
| (Perez et al., 2017) | | No sex-specific aspects | Metabolism |
| (Canterini et al., 2018) | No sex-specific aspects? | | Eating disorders Mental disorders |
| (Freese et al., 2017) | | | Mental disorders |
| (Lian et al., 2017) | | | Urinary incontinence / Pelvic floor disorders |
| (Resende et al., 2018) | | No sex-specific aspects | (Epigenetic aspects) |
| (Sanmiguel et al., 2017) | | No sex-specific aspects | (Microbiome / mental disorders) |
| (Kurnicka et al., 2018) | | No sex-specific aspects | (Heart / cardiovascular disorders) |
| (Escobar-Morreale et al., 2017) | | | Sexual hormones / PCOS |
| (Jesús M. Gómez-Martin et al., 2017) | | No sex-specific aspects | (Heart / cardiovascular disorders) |
| (Onofre et al., 2017) | | No sex-specific aspects | (Lungs function / disorders) |
| (Stuart & Källen, 2017) | | | Pregnancy |
| (Chiofalo et al., 2017) | | | Sexual hormones / PCOS |
| (Kim et al., 2017) | | | Urinary incontinence / Pelvic floor disorders |
| (Milone et al., 2017) | | | Infertility/ pregnancy |
| (Martinez de la Escalera et al., 2017) | | No sex-specific aspects | (Microbiome) |
| (Seo, Lee, Torabi, & Lohrmann, 2017) | | | Outcome/ Follow-up |
| (Dolin, Ude Welcome, & Caughey, 2016) | | | Pregnancy |
| (Steffen et al., 2017) | | | Sexuality/ sexual function |
| (Vrbikova et al., 2016) | | No sex-specific aspects | Diabetes |
| (Lopes Gomes et al., 2017) | | No sex-specific aspects | (Nutrition/ supplementation) |
| (Parent et al., 2017) | | | Pregnancy / Childbearing |
| (Zmolikova et al., 2016) | No sex-specific aspects? | | Mental disorders |
| (Neovius & Stephansson, 2016) | | | Pregnancy / Childbearing |
| (Hessami, Azar, Shklyanka, & Trivedi, 2015) | | | Urinary incontinence / Pelvic floor disorders |

| | | | |
|---|---|---|---|
| (Jans et al., 2016) | | | Pregnancy / postpartum period/ epidemiology |
| (Edison et al., 2016) | | | Pregnancy / fertility |
| (Bhandari et al., 2016) | | | Sexual hormones / PCOS |
| (B. Mengesha et al., 2016) | | | Contraception |
| (de Barros et al., 2016) | | No sex-specific aspects | (Non-alcoholic fatty liver disease) |
| (Abenhaim, Alrowaily, Czuzoj-Shulman, Spence, & Klam, 2016) | | | Pregnancy |
| (Kunesova et al., 2015) | | No sex-specific aspects | (Diabetes) |
| (Moreno-Castellanos et al., 2016) | | No sex-specific aspects | (Diabetes) |
| (Kumari & Nigam, 2015) | | | Pregnancy |
| (Delgado Floody, 2015) | | No sex-specific aspects | (Therapeutic concept) |
| (O'Boyle, O'Sullivan, Shabana, Boyce, & O'Reilly, 2016b) | | | Urinary incontinence / Pelvic floor disorders |
| (Grong et al., 2016) | | | Hormone secretion |
| (Gomes, de Almeida Oliveira, Dutra, Pizato, & de Carvalho, 2016) | | No sex-specific aspects | (Nutrition / Body composition) |
| (Goughnour et al., 2016) | | | Menopausal symptoms |
| (Robson, Daniels, & Rawlings, 2016) | Only comment / no research article | | Pregnancy |
| (Monshi et al., 2015) | | | Breastfeeding/ dermatology |
| (Costa Justus et al., 2016) | | No sex-specific aspects | (Endocrinology) |
| (Shimonov, Groutz, Schachter, & Gordon, 2017b) | | | Urinary incontinence / Pelvic floor disorders |
| (Menegati et al., 2016) | | | Menopause / bone health |
| (Sanguankeo & Upala, 2016) | Only comment / no research article | | Endometrial cancer |
| (Lamy et al., 2015) | | No sex-specific aspects | (Eating behavior) |
| (Sanguankeo & Upala, 2016) | Only comment / no research article | | (Endometrial cancer) |
| (Carpio, 2015) | | No sex-specific aspects | (Lungs and OSAS) |
| (Neff et al., 2015) | | | endometrial cancer / quality of life |

| | | | |
|---|---|---|---|
| (Needleman & Noria, 2015) | Only comment / no research article | | Outcome |
| (Parker, Berghella, & Nijjar, 2016) | | | Pregnancy |
| (L. B. R. de Souza, Pernambuco, dos Santos, & Pereira, 2016) | (No sex-specific aspects?) | | Vocation |
| (Sánchez et al., 2016) | | | Osteoporosis |
| (Modesitt et al., 2015) | | | Endometrial cancer |
| (Silva et al., 2015) | | No sex-specific aspects | (Epigenetics) |
| (Benito et al., 2015) | Case report | | Endometrial cancer |
| (Coker, von Lojewski, Luscombe, & Abraham, 2015) | | No sex-specific aspects | (Eating behavior) |
| (Ogle, Park, Damhorst, & Bradley, 2016) | | | Social aspects |
| (Marques de Oliveira, 2015) | | No sex-specific aspects | (Chronic inflammation/ micronutritients) |
| (Fereidouni et al., 2015) | | | Mental disorders |
| (Kennedy-Dalby et al., 2014) | | | Outcome |
| (Ramalho et al., 2015) | | | Sexuality / sexual function / skin |
| (Toro-Ramos et al., 2015) | | No sex-specific aspects | (Adipose tissue / body composition) |
| (Casas et al., 2014) | | | Contraception |
| (Lecube et al., 2015) | | No sex-specific aspects | (Micronutrition) |
| **MEDLINE Bariatric AND Female** | | | |
| (Lu et al., 2019) | | | Mental disorders |
| (Ciangura et al., 2019) | | | Pregnancy / childbearing |
| (Sockalingam et al., 2019) | | | Mental disorders |
| (Wein Alan J., 2018) | | Only editorial comment | |
| (Gasa et al., 2019) | | No sex-specific aspects | (Sleep and OSAS) |
| (Lechmiannandan et al., 2019) | | Duplicate | |
| (Perdue, Schreier, Swanson, Neil, & Carels, 2018b) | | | Mental disorders |
| (Herrick, Favela, Simerly, Abumrad, & Bingham, 2018) | | No sex-specific aspects | Hypothalamus / glia |
| (Breffini Anglim et al., 2018a) | | | Urinary incontinence / Pelvic floor disorders |
| (Perdue, Schreier, Swanson, Neil, & Carels, 2018a) | | Duplicate | |
| (Leshem, Shimonov, Amir, Gordon, & Groutz, 2017b) | | | Urinary incontinence / Pelvic floor disorders |
| (Anveden et al., 2017) | | | (Endometrial) cancer |
| (Charalampakis et al., 2016) | | | Sexual hormones/ PCOS |

| | | | |
|---|---|---|---|
| (Janse Van Vuuren, Strodl, White, & Lockie, 2018) | | | Mental disorders |
| (Seftel Allen D., 2016) | | Answer/ Comment | Urinary incontinence / Pelvic floor disorders |
| (Babarina, Fadeeva, Savelyeva, & Khallaeva, 2015) | | No trustworthy publication / no doi / case report in Russian, abstract without information | |
| (Young et al., 2016) | | | Outcome |
| (Knepfler et al., 2016b) | | | Urinary incontinence / Pelvic floor disorders |
| (Dikareva, Harvey, Cicchillitti, Bartlett, & Andersen, 2016) | | | Physical activity (mental health) |
| (Janik et al., 2015) | | | Sexuality/ Sexual function |
| (Benediktsdottir, Halldorsson, Bragadottir, Gudmundsson, & Ramel, 2016) | | | Mental disorders |
| **MEDLINE Bariatric AND Sex** | | | |
| (Casimiro et al., 2019) | | | Sexual hormons /PCOS Menopause/ osteoporosis |
| (Lee et al., 2019) | | Only affects men | |
| (Sarwer et al., 2018) | | Duplicate | |
| (Grayson et al., 2017) | | | Metabolism / liver |
| (Boonchaya-anant et al., 2016) | | Only affects men | |
| (Bardisi et al., 2016) | | Only affects men | |
| (Afarideh et al., 2016) | Comment, refers to included article | | Outcome |
| (Wee et al., 2016) | | | Quality of life |
| (Cunha et al., 2016) | | No sex-specific aspects | (Outcome) |
| (Sarwer et al., 2015) | | Only affects men | |
| (Reis, 2015) | | Only affects men | |
| **MEDLINE Bariatric AND Gender** | | | |
| (Duarte-Guerra, Coêlho, Santo, Lotufo-Neto, & Wang, 2018) | | | Mental disorders |
| (Biörserud et al., 2018) | | | Mental disorders/ Skin |
| (Hiwa O. Ahmed et al., 2018) | | | Plastic surgery |
| (Horvath et al., 2018) | | | Sleep / OSAS |
| (Kochkodan et al., 2018) | | | Outcome /weight loss / quality of life |
| (Stroh et al., 2015) | Comment | | Outcome / Comorbidities |

| | | |
|---|---|---|
| (Miller-Matero, Tobin, Clark, Eshelman, & Genaw, 2016) | | Mental disorders |
| (Applegate, 2015) | Comment, does not refer to included article | Instruments |
| (Marek, Ben-Porath, Sellbom, McNulty, & Heinberg, 2015) | | Instruments |
| **Academic Search Premier (EBSCO) Bariatric AND Women OR female OR woman OR females** | | |
| (Abraham, Neha, & Power, 2019) | | Pregnancy |
| (Wiemerslage et al., 2017) | No sex-specific aspects | (Neuroscience) |
| (Perdue et al., 2018a) | Duplicate | |
| (Garretto et al., 2019) | Duplicate | |
| (Janse Van Vuuren et al., 2018) | Duplicate | |
| (Al kabbi et al., 2018) | | Fertility |
| (Newlan & Geraghty, 2018) | | Pregnancy |
| (Bhandari et al., 2016) | Duplicate | |
| (Gasa et al., 2019) | Duplicate | |
| (Vincentelli et al., 2018) | Duplicate | |
| (Harreiter et al., 2018) | Duplicate | |
| (Breffini Anglim et al., 2018a) | Duplicate | |
| (خسروی, امیی, پورسریی, سبحانی, & صادقیان, 2018) | No sex-specific aspects, authors and affiliation only in Persian | |
| (Anveden et al., 2017) | Duplicate | |
| (Martinez de la Escalera et al., 2017) | Duplicate | |
| (Pansani Maniglia, Grundmann Grundmann, & da Silva, 2018) | No sex-specific aspects | |
| (Perez et al., 2017) | Duplicate | |
| (Hazart et al., 2017) | Duplicate | |
| (J. M. Gómez-Martin et al., 2018) | Duplicate | |
| (Moore, Chandler, Holland, Davis-Smith, & King, 2017) | | Social aspects |
| (Michalsky et al., 2019) | | Contraception and fertility |
| (Goughnour et al., 2016) | Duplicate | |
| (Bartholomay et al., 2019) | Duplicate | |
| (Jans, Devlieger, et al., 2018) | | Pregnancy and breast-feeding |
| (Uruç et al., 2016) | | Urinary incontinence and pelvic floor disorders |
| (Zmolikova et al., 2016) | Duplicate | |
| (Mallipedhi et al., 2015) | | Metabolism |
| (Whyte et al., 2016) | | Pregnancy |
| (Leshem et al., 2017b) | Duplicate | |

| | | |
|---|---|---|
| (Ogle et al., 2016) | Duplicate | |
| (Kumari & Nigam, 2015) | Duplicate | |
| (Herrick et al., 2018) | Duplicate | |
| (Neff et al., 2015) | Duplicate | |
| (Modesitt et al., 2015) | Duplicate | |
| (Silva et al., 2015) | Duplicate | |
| (Coker et al., 2015) | Duplicate | |
| (Kennedy-Dalby et al., 2014) | Duplicate | |
| (Lian et al., 2017) | Duplicate | |
| (Charalampakis et al., 2016) | Duplicate | |
| (Escobar-Morreale et al., 2017) | Duplicate | |
| (Stuart & Källen, 2017) | Duplicate | |
| (Dikareva et al., 2016) | Duplicate | |
| (Galazis, Docheva, Simillis, & Nicolaides, 2014) | | Pregnancy |
| (Abenhaim et al., 2016) | Duplicate | |
| (Young et al., 2016) | Duplicate | |
| (Palma et al., 2018) | Duplicate | |
| (Jans et al., 2016) | Duplicate | |
| (B. Mengesha et al., 2016) | Duplicate | |
| (Parker et al., 2016) | Duplicate | |
| (K. McLean, Moore, Miketinas, & Champagne, 2017) | No sex specific aspects? | (Eating behaviour) |
| (Robson et al., 2016) | Duplicate | |
| (Delgado Floody, 2015) | Duplicate | |
| (Maric et al., 2017) | | Pregnancy |
| (Carpio, 2015) | Duplicate | |
| (Marques de Oliveira, 2015) | Duplicate | |
| (Nimbi et al., 2017) | | Sexuality and sexual function |
| (Brewer, 2017) | | Female-specific cancer |
| (Needleman & Noria, 2015) | Duplicate | |
| (Hoel, 2015) | | Female-specific cancer |
| (J. Christ & Falcone, 2016) | | Sexual hormons and PCOS |
| (Young et al., 2016) | Duplicate | |
| (Jernigan et al., 2016a) | | Female-specific cancer |
| (Jernigan et al., 2016b) | | Female-specific cancer |
| (Muniz Magalhaes, Gois Campos, Sarin, & Del Porto, 2016) | | Mental disorders(Nimbi et al., 2017) |
| (Nimbi et al., 2017) | Duplicate | |
| („Clinical digest. Significantly more women than men have bariatric surgery as trend continues“, 2015) | No authors, source seems not to be trustworthy | |

| | | |
|---|---|---|
| **Academic Search Premier (EBSCO) Bariatric AND Sex Or Gender** | | |
| (Biörserud et al., 2018) | Duplicate | |
| (Duarte-Guerra et al., 2018) | Duplicate | |
| (Casimiro et al., 2019) | Duplicate | |
| (Casimiro et al., 2019) | Duplicate | |
| (Grayson et al., 2017) | Duplicate | |
| (Bardisi et al., 2016) | Duplicate | |
| (Boonchaya-anant et al., 2016) | Duplicate | |
| (Kochkodan et al., 2018) | Duplicate | |
| (Camacho-Laraña, Alcalá-Pérez, & Nieves-Alcalá, 2015) | | Outcome |
| (Afarideh et al., 2016) | Duplicate | |
| **PsycInfo (EBSCO): Bariatric AND women OR female OR woman OR females** | | |
| (Couch, 2018) | | Mental disorders |
| (Kinville, 2018) | | Mental disorders |
| (Sanmiguel et al., 2017) | Duplicate | |
| (Moore et al., 2017) | Duplicate | |
| (Freese et al., 2017) | Duplicate | |
| (Zmolikova et al., 2016) | Duplicate | |
| (Ogle et al., 2016) | Duplicate | |
| (Obetsanov, 2016) | Duplicate | |
| (Fereidouni et al., 2015) | Duplicate | |
| (Coker et al., 2015) | Duplicate | |
| (Dawson, 2015) | | Mental disorders |
| (Konik & Smith, 2015) | No scientific publication / no peer-reviewed journal | |
| (Gasa et al., 2019) | Duplicate | |
| (Zayed, 2019) | | Social aspects |
| (Perdue et al., 2018b) | Duplicate | |
| (Perdue et al., 2018a) | Duplicate | |
| (Wiemerslage et al., 2017) | Duplicate | |
| (Dikareva et al., 2016) | Duplicate | |
| **PsycInfo (EBSCO) Bariatric AND sex OR gender** | | |
| (Duarte-Guerra et al., 2018) | Duplicate | |
| (Camacho-Laraña et al., 2015) | Duplicate | |
| **Cochrane Library (only Reviews and Protocols) Bariatric** | | |
| (Kitson et al., 2018) | | Female specific cancer |
| (Dong et al., 2016) | Out of topic | |
| (Piper et al., 2015) | | Else (idiopatic tntracranial hypertension) |

| | | | |
|---|---|---|---|
| (Opray, Grivell, Deussen, & Dodd, 2015) | | | Pregnancy |
| (Colquitt, Pickett, Loveman, & Frampton, 2014) | | No sex-specific aspects! | |
| (Ells et al., 2015) | | No sex-specific aspects! | |
| (Chavez-Tapia et al., 2010) | | | |
| (Jefferys, Siassakos, Draycott, Akande, & Fox, 2013) | | | Pregnancy |
| (Parra, Riera, Atallah, & Porfirio, 2014) | | No sex-specific aspects | |
| (Fernandez-Montoli et al., 2018) | | | Female specific cancer |
| (Chakhtoura et al., 2015) | | No sex-specific aspects! | |
| (G. M. Souza, Santos, Barbosa, & Melnik, 2018) | | Withdrawn from the publisher | |
| (Morandeira-Rivas et al., 2018) | | Withdrawn from the publisher | |
| **Scopus: bariatric AND women OR female OR sex OR gender** | | | |
| (Koehler et al., 2019) | | Duplicate | |
| (Maslin et al., 2019) | | Duplicate | |
| (Abraham et al., 2019) | | Duplicate | |
| (Lu et al., 2019) | | Duplicate | |
| (Shah et al., 2019) | | Duplicate | |
| (Hult, Bonn, Brandt, Wirén, & Lagerros, 2019) | Sex-specific aspects? | | Quality of life |
| (Nogué et al., 2019) | | Duplicate | |
| (Zeller et al., 2019) | | | Sexuality and sexual function |
| (Steffen et al., 2017) | | Duplicate | |
| (Steffen et al., 2017) | | Duplicate | |
| (Lechmiannandan et al., 2019) | | Duplicate | |
| (Cherick et al., 2019) | | Duplicate | |
| (Casimiro et al., 2019) | | Duplicate | |
| (Reichmann et al., 2019) | | No sex-specific aspects | |
| (Menke et al., 2019) | | Duplicate | |
| (Hubert et al., 2019) | | Duplicate | |
| (Vieira et al., 2019) | | Duplicate | |
| (B. Anglim, O'Boyle, O'Sullivan, & O'Reilly, 2019) | | Only note, not available, no abstract | |
| (C. F. de A. Oliveira et al., 2018) | | | Sexuality and sexual function |
| (Lee et al., 2019) | | Duplicate | |
| (Beato et al., 2019b) | | Duplicate | |
| (Beato et al., 2019a) | | Duplicate | |
| (Faria et al., 2019) | | Duplicate | |
| (Sockalingam et al., 2019) | | Duplicate | |

| | | | |
|---|---|---|---|
| (Gasa et al., 2019) | | Duplicate | |
| (Damhof et al., 2019) | | Duplicate | |
| (Luhrs et al., 2019) | | Duplicate | |
| (Sande-Lee et al., 2019) | | Duplicate | |
| (Balestrin et al., 2019) | | Duplicate | |
| (Dereppe et al., 2019) | | Duplicate | |
| (Pichlerova et al., 2019) | | Duplicate | |
| (Breffini Anglim et al., 2018a) | | Duplicate | |
| (Al kabbi et al., 2018) | | Duplicate | |
| (Duarte-Guerra et al., 2018) | | Duplicate | |
| (Perdue et al., 2018a) | | Duplicate | |
| (Newlan & Geraghty, 2018) | | Duplicate | |
| (Abiad et al., 2018) | | Duplicate | |
| (Herrick et al., 2018) | | Duplicate | |
| (Pinhel et al., 2018) | | Duplicate | |
| (Biörserud et al., 2018) | | Duplicate | |
| (Horvath et al., 2018) | | Duplicate | |
| (Janse Van Vuuren et al., 2018) | | Duplicate | |
| (Hiwa O. Ahmed et al., 2018) | | Duplicate | |
| (J. P. Christ & Falcone, 2018) | | Duplicate | |
| (Jans, Devlieger, et al., 2018) | | Duplicate | |
| (Soares Júnior et al., 2018) | | | Fertility |
| (B. M. Mengesha et al., 2018) | | Duplicate | |
| (Graham et al., 2018) | | Duplicate | |
| (Vincentelli et al., 2018) | | Duplicate | |
| (J. M. Gómez-Martin et al., 2018) | | Duplicate | |
| (Palma et al., 2018) | | Duplicate | |
| (Jans, Matthys, et al., 2018) | | Duplicate | |
| (Sarwer et al., 2018) | | Duplicate | |
| (Ficaro, 2018) | | Duplicate | |
| (Gimenes et al., 2018) | | Duplicate | |
| (Kochkodan et al., 2018) | | Duplicate | |
| (Ravelli et al., 2018) | Sex specific aspects? | | Mental aspects |
| (Gabriel et al., 2018b) | | Duplicate | |
| (Kurnicka et al., 2018) | | Duplicate | |
| (Wein Alan J., 2018) | | Duplicate | |
| (Canterini et al., 2018) | | Duplicate | |
| (Perdue et al., 2018b) | | Duplicate | |
| (Al-Shaikh et al., 2018a) | | | Urinary incontinence/ pelvic floor disorders |
| (Garretto et al., 2019) | | Duplicate | |
| (Leclercq et al., 2018) | | | Pregnancy |
| (Harreiter et al., 2018) | | Duplicate | |
| (de Campos et al., 2018) | | Duplicate | |
| (K. L. McLean et al., 2018) | | Duplicate | |
| (H.O. Ahmed, 2017) | | | Fertility |
| (Seftel, 2017) | | | Urinary incontinence/ pelvic floor disorders |

| | | | |
|---|---|---|---|
| (TERRA et al., 2017) | | Duplicate | |
| (Sanmiguel et al., 2017) | | Duplicate | |
| (Perez et al., 2017) | | Duplicate | |
| (Schnor et al., 2017) | | Duplicate | |
| (Lian et al., 2017) | | Duplicate | |
| (Onofre et al., 2017) | | Duplicate | |
| (Milone et al., 2017) | | Duplicate | |
| (Leshem et al., 2017b) | | Duplicate | |
| (Escobar-Morreale et al., 2017) | | Duplicate | |
| (Moore et al., 2017) | | Duplicate | |
| (Chiofalo et al., 2017) | | Duplicate | |
| (Freese et al., 2017) | | Duplicate | |
| (Anveden et al., 2017) | | Duplicate | |
| (Jesús M. Gómez-Martin et al., 2017) | | Duplicate | |
| (Kim et al., 2017) | | Duplicate | |
| (Seo et al., 2017) | | Duplicate | |
| (Martinez de la Escalera et al., 2017) | | Duplicate | |
| (Wiemerslage et al., 2017) | | Duplicate | |
| (Parent et al., 2017) | | Duplicate | |
| (Steffen et al., 2017) | | Duplicate | |
| (Lopes Gomes et al., 2017) | | Duplicate | |
| (Grayson et al., 2017) | | Duplicate | |
| (Figueiredo, Lourdes, Machado, & Conceição, 2017) | No peer-reviewed article? | | Mental aspects |
| (Melo & Melo, 2017) | | | Fertility |
| (Hazart et al., 2017) | | Duplicate | |
| (Stuart & Källen, 2017) | | Duplicate | |
| (Vrbikova et al., 2016) | | Duplicate | |
| (Shimonov et al., 2017b) | | Duplicate | |
| (Charalampakis et al., 2016) | | Duplicate | |
| (Dolin et al., 2016) | | Duplicate | |
| (Neovius & Stephansson, 2016) | | Duplicate | |
| (Edison et al., 2016) | | Duplicate | |
| (Abenhaim et al., 2016) | | Duplicate | |
| (Bardisi et al., 2016) | | Duplicate | |
| (Bhandari et al., 2016) | | Duplicate | |
| (Zmolikova et al., 2016) | | Duplicate | |
| (Dikareva et al., 2016) | | Duplicate | |
| (de Barros et al., 2016) | | Duplicate | |
| (Afarideh et al., 2016) | | Duplicate | |
| (Moreno-Castellanos et al., 2016) | | Duplicate | |
| (Jans et al., 2016) | | Duplicate | |
| (B. Mengesha et al., 2016) | | Duplicate | |
| (O'Boyle et al., 2016b) | | Duplicate | |
| (Grong et al., 2016) | | Duplicate | |
| (Gomes et al., 2016) | | Duplicate | |
| (Menegati et al., 2016) | | Duplicate | |
| (Parker et al., 2016) | | Duplicate | |
| (Wee et al., 2016) | | Duplicate | |

| | | |
|---|---|---|
| (Whyte et al., 2016) | Duplicate | |
| (Cunha et al., 2016) | Duplicate | |
| (Mahmood & Thanoon, 2016) | | Fertility |
| (Knepfler et al., 2016b) | Duplicate | |
| (Young et al., 2016) | Duplicate | |
| (L. B. R. de Souza et al., 2016) | Duplicate | |
| (Seftel, 2017) | Duplicate | |
| (Costa Justus et al., 2016) | Duplicate | |
| (Sánchez et al., 2016) | Duplicate | |
| (Goughnour et al., 2016) | Duplicate | |
| (Miller-Matero et al., 2016) | Duplicate | |
| (Boonchaya-anant et al., 2016) | Duplicate | |
| (Benediktsdottir et al., 2016) | Duplicate | |
| (D. M. de Oliveira et al., 2016) | | Mental aspects |
| (Sanguankeo & Upala, 2016) | Duplicate | |
| (Robson et al., 2016) | Duplicate | |
| (Upala & Sanguankeo, 2016) | Duplicate | Female specific cancer |
| (Ogle et al., 2016) | Duplicate | |

| **Additional sources identified through hand search** | | |
|---|---|---|
| (Rosen et al., 2000) | | |
| (Schlatter, 2017) | | contraceptives |
| (Ginstman et al., 2019) | | contraceptives |
| (Merki-Feld et al., 2015) | | Contraceptives |
| (Curtis et al., 2016) | | contraceptives |
| (Guttmacher Institute, 2004) | | contraceptives |
| (World Health Organization (WHO), 2018) | | contraceptives |
| (Ined - Institut national d'études démographiques, 2017) | | contraceptives |
| (Durlinger et al., 2002) | | fertility |
| (Grynnerup et al., 2012) | | fertility |
| (Gambineri et al., 2019) | | fertility |
| (Sam, 2007) | | PCOS |
| (Achari & Jain, 2017) | | PCOS, hormones |
| (van Wijk et al., 2016) | | PCOS, hormones |
| (Upala & Anawin Sanguankeo, 2015) | | cancer |
| (Wright et al., 2017) | | osteoporosis |
| (Ji & Yu, 2015) | | osteoporosis |
| (Smid et al., 2017) | | Pregnancy/ breastfeeding |
| (Gadgil et al., 2014) | | Pregnancy/ breastfeeding |
| (Guelinckx, Devlieger, Beckers, & Vansant, 2008) | | Pregnancy/ breastfeeding |

| | |
|---|---|
| (Stothard, Tennant, Bell, & Rankin, 2009) | Pregnancy/ breastfeeding |
| (Bellamy, Casas, Hingorani, & Williams, 2009) | Pregnancy/ breastfeeding |
| (McCall, Li, Kurinczuk, Sullivan, & Knight, 2019) | Pregnancy/ breastfeeding |
| (Shawe et al., 2019) | Pregnancy/ breastfeeding |

**Table S2: Screened records of systematic literature search and assignment into categories**

*The records of the systematic literature search are presented in the assigned categories. Sources that are used in several categories are highlighted in blue. Notes and reasons for exclusion are listed in the middle column.*

| | | |
|---|---|---|
| **Contraception** | | |
| (Shah et al., 2019) | | contraception |
| (Damhof et al., 2019) | | contraception |
| (Graham et al., 2018) | | contraception |
| (Mengesha et al., 2018) | | contraception |
| (Mengesha et al., 2016) | | contraception |
| (Casas et al., 2014) | | contraception |
| (Michalsky et al., 2019) | | Contraception and fertility |
| (Nimbi et al., 2017) | | Contraception /Sexuality and sexual function |
| **Fetility/ Infertility** | | |
| (Menke et al., 2019) | | infertility |
| (Vincentelli et al., 2018) | | Fertility/AMH |
| (Milone et al., 2017) | | Infertility (pregnancy) |
| (Edison et al., 2016) | | Pregnancy / fertility |
| (Al kabbi et al., 2018) | | fertility |
| (Soares Júnior et al., 2018) | | Fertility |
| (Ahmed, 2017) | | fertility |
| (Melo and Melo, 2017) | Excluded, abstract without information, article in Spanish | Fertility |
| (Mahmood and Thanoon, 2016) | Excluded, abstract without sufficient information | Fertility |
| (Chiofalo et al., 2017) | | Sexual hormons / PCOS -AMH (fertility) |
| (Bhandari et al., 2016) | | Sexual hormons / PCOS |
| (Charalampakis et al., 2016) | | Sexual hormons/ PCOS |
| **Pregnancy & breastfeeding** | | |
| (Maslin et al., 2019) | - | pregnancy |
| (Bartholomay et al., 2019) | | Pregnancy /vitamins |
| (Balestrin et al., 2019) | | pregnancy |
| (Garretto et al., 2019) | | Pregnancy/ breastfeeding |
| (Faria et al., 2019) | | Pregnancy/ adherence |
| (Harreiter et al., 2018) | | pregnacy |
| (Jans et al., 2018b) | | Pregnancy (mental disorders) |
| (Hazart et al., 2017) | | Pregnancy / Child bearing |
| (Gimenes et al., 2018) | | Pregnancy / Child bearing/ Breastfeeding |
| (Stuart and Källen, 2017) | | Pregnancy / follow procedures |
| (Dolin et al., 2016) | | pregnancy |
| (Parent et al., 2017) | | Pregnancy / Child bearing |
| (Neovius and Stephansson, 2016) | | Pregnancy / Child bearing |

| | | |
|---|---|---|
| (Jans et al., 2016) | | Pregnancy / post partum epidemiology |
| (Edison et al., 2016) | | Pregnancy / fertility |
| (Abenhaim et al., 2016) | | pregnancy |
| (Kumari and Nigam, 2015) | | pregnancy |
| (Robson et al., 2016) | Only commentary | pregnancy |
| (Monshi et al., 2015) | | Breastfeeding/ dermatology |
| (Parker et al., 2016) | | pregnancy |
| (Ciangura et al., 2019) | | Pregnancy / childbearing |
| (Abraham et al., 2019) | | pregnancy |
| (Newlan and Geraghty, 2018) | | pregnancy |
| (Jans et al., 2018a) | | Pregnancy and brest-feeding |
| (Whyte et al., 2016) | | pregnancy |
| (Galazis et al., 2014) | | pregnancy |
| (Maric et al., 2017) | | pregnancy |
| (Opray et al., 2015) | | pregnancy |
| (Leclercq et al., 2018) | | pregnancy |
| **Pelvic floor disorders and urinary incontinece** | | |
| (Keller et al., 2019) | | Quality of live<br>Urinary incontinence |
| (Paka et al., 2019) | | Urinary incontinence |
| (Zhang et al., 2018) | | Urinary incontinence |
| (Gabriel et al., 2018) | | Urinary incontinence |
| (Lian et al., 2017) | | Urinary incontinence / Pelvic floor disorders |
| (Kim et al., 2017) | | Urinary incontinence / Pelvic floor disorders |
| (Hessami et al., 2015) | No meaningful English abstract available | Urinary incontinence / Pelvic floor disorders |
| (O’Boyle et al., 2016) | | Urinary incontinence / Pelvic floor disorders |
| (Shimonov et al., 2017) | | Urinary incontinence / Pelvic floor disorders |
| (Anglim et al., 2018) | | Urinary incontinence / Pelvic floor disorders |
| (Leshem et al., 2017) | | Urinary incontinence / Pelvic floor disorders |
| (Seftel Allen D., 2016) | Answer/ Comment, no abstract available | Urinary incontinence / Pelvic floor disorders |
| (Knepfler et al., 2016) | | Urinary incontinence / Pelvic floor disorders |
| (Uruç et al., 2016) | | Urinary incontinence and pelvic floor disorders |
| (Al-Shaikh et al., 2018) | | Urinary incontinence/ pelvic floor disorders |
| (Seftel, 2017) | Answer to a comment, no abstract available | Urinary incontinence/ pelvic floor disorders |
| **Female specific cancer** | | |
| (Ganesan and Choy, 2019) | Only comment / no research article | (breast cancer) |

| | | |
|---|---|---|
| (Feigelson et al., 2019b) | Only response to comment / no research article | (breast cancer) |
| (Feigelson et al., 2019a) | | Breast cancer |
| (Neff et al., 2015) | | endometrial cancer / quality of life |
| (Sanguankeo and Upala, 2016) | Comment | (endometrial cancer) |
| (Modesitt et al., 2015) | | Endometrial cancer /quality of life |
| (Benito et al., 2015) | Casuistic | Endometrial cancer |
| (Anveden et al., 2017) | | (endometrial) cancer |
| (Brewer, 2017) | No number of cases or follow-up period in abstract available; no reply of the contacted authors | Female-specific cancer |
| (Hoel, 2015) | | Female-specific cancer |
| (Jernigan et al., 2016a) | | Female-specific cancer |
| (Jernigan et al., 2016b) | No sex-specific aspects | Female-specific cancer |
| (Kitson et al., 2018) | | female specific cancer |
| (Fernandez-Montoli et al., 2018) | | female specific cancer |
| (Upala and Sanguankeo, 2016) | Comment/ Answer | Female specific cancer |
| (Charalampakis et al., 2016) | | Sexual hormons/ PCOS + endometrial hyperplasy |
| (Needleman and Noria, 2015) | Comment | (outcome?) |
| (Wee et al., 2016) | | Quality of life |
| (Upala and Sanguankeo, 2015) | | female specific cancer |
| **Menopause and osteoporosis** | | |
| (Luhrs et al., 2019) | | osteoporosis |
| (Goughnour et al., 2016) | | Menopausal symptoms |
| (Menegati et al., 2016) | | Menopause / bone health |
| (Sánchez et al., 2016) | | osteoporosis |
| (Casimiro et al., 2019) | | Sexual hormons /PCOS Menopause/ osteoporosis |
| **Sexuality and sexual function** | | |
| (Pichlerova et al., 2019) | | Sexuality/sexual function |
| (Steffen et al., 2019) | | Sexuality/sexual function |
| (Lechmiannandan et al., 2019) | | Sexuality/sexual function |
| (Sarwer et al., 2018) | | Sex hormons Quality of life Sexuality/sexual function |
| (Steffen et al., 2017) | | Sexuality/sexual function |
| (Ramalho et al., 2015) | | Sexuality / sexual function / skin |
| (Janik et al., 2015) | | Sexuality/ Sexual function |
| (Zeller et al., 2019) | | Sexuality and sexual function |
| (Oliveira et al., 2018) | | Sexuality and sexual function |
| (Cherick et al., 2019) | | Quality of life & Self-esteemed health |
| **Sex hormons and PCOS** | | |
| (Abiad et al., 2018) | | Sex hormons/ PCOS |
| (Christ and Falcone, 2018) | | Sex hormons/PCOS |

| | | |
|---|---|---|
| (Sarwer et al., 2018) | | Sex hormons<br>Quality of life<br>Sexuality/sexual function |
| (Escobar-Morreale et al., 2017) | | Sexual hormons / PCOS |
| (Bhandari et al., 2016) | | Sexual hormons / PCOS |
| (Charalampakis et al., 2016) | | Sexual hormons/ PCOS |
| (Casimiro et al., 2019) | Educational paper | Sexual hormons /PCOS<br>Menopause/ osteoporosis |
| (Christ and Falcone, 2016) | | Sexual hormons and PCOS |
| (Graham et al., 2018) | | PCOS & contraception |
| **Social aspects** | | |
| (Ficaro, 2018) | | Social aspects |
| (Ogle et al., 2016) | | Social aspects |
| (Moore et al., 2017) | | Social aspects |
| (Zayed, 2019) | | Social aspects |
| **Mental health / mental disorders** | | |
| (Canterini et al., 2018) | (No sex-specific aspects) | Eating disorders<br>Mental disorders |
| (Freese et al., 2017) | | Mental disorders |
| (Zmolikova et al., 2016) | | Mental disorders () |
| (Fereidouni et al., 2015) | | Mental disorders |
| (Lu et al., 2019) | | Mental disorders |
| (Sockalingam et al., 2019) | | Mental disorders |
| (Perdue et al., 2018) | | Mental disorders |
| (Janse Van Vuuren et al., 2018) | | Mental disorders |
| (Dikareva et al., 2016) | | Physical activity (mental health) |
| (Benediktsdottir et al., 2016) | | Mental disorders |
| (Duarte-Guerra et al., 2018) | | Mental disorders |
| (Biörserud et al., 2018) | | Mental disorders/ Skin |
| (Miller-Matero et al., 2016) | | Mental disorders |
| (Muniz Magalhaes et al., 2016) | | Mental disorders(Nimbi et al., 2017) |
| (Couch, 2018) | | Mental disorders |
| (Kinville, 2018) | | Mental disorders |
| (Dawson, 2015) | | Mental disorders |
| (Ravelli et al., 2018) | (No sex specific aspects?) | Mental disorders |
| (Figueiredo et al., 2017) | (No peer-reviewed article..?) | Mental disorders |
| (de Oliveira et al., 2016) | | Mental disorders |
| **Eating behaviour and disorders** | | |
| (Nogué et al., 2019) | (No sex-specific aspects?) | (Eating behavior/ disorders) |
| (Reichmann et al., 2019) | (No sex-specific aspects?) | (Eating behavior/ disorders) |
| (Hubert et al., 2019) | (No sex-specific aspects?) | (Eating behavior/ disorders) |
| (Vieira et al., 2019) | (No sex-specific aspects?) | (Eating behavior/ disorders) |
| (Canterini et al., 2018) | (No sex-specific aspects?) | Eating disorders<br>Mental disorders |
| (Coker et al., 2015) | (No sex-specific aspects?) | (eating behavior) |
| (McLean et al., 2017) | No abstract available | (eating behaviour) |

| **Metabolism, Outcome/ Follow-Up / quality of life** | | |
|---|---|---|
| (Seo et al., 2017) | No sex-specific aspects, excluded | Follow-up () |
| (Kennedy-Dalby et al., 2014) | | outcome |
| (Young et al., 2016) | | outcome |
| (Afarideh et al., 2016) | Comment refers to used article | outcome |
| (Kochkodan et al., 2018) | | Outcome /weight loss / quality of life |
| (Camacho-Laraña et al., 2015) | Only in Spain, excluded | outcome |
| (Keller et al., 2019) | | Quality of live<br>Urinary incontinence |
| (Hult et al., 2019) | No sex-specific aspects, excluded | Quality of life |
| (Grayson et al., 2017) | | Metabolism / liver |
| (Mallipedhi et al., 2015) | No sex-specific aspects, excluded | Metabolism |
| (Grong et al., 2016) | No sex-specific aspects, excluded | Hormone secretion |
| (Stroh et al., 2015) | | Outcome |
| **Else** | | |
| (de Souza et al., 2016) | No sex-specific aspects, excluded | vocation |
| (Horvath et al., 2018) | | Sleep / OSAS |
| (Marek et al., 2015) | No sex-specific aspects, excluded | Instruments |
| (Piper et al., 2015) | Bariatric surgery not discussed | Else (idiopatic tntracranial hypertension) |
| (Biörserud et al., 2018) | | Mental disorders/ Skin |
| (Ahmed et al., 2018) | | Plastic surgery |

**Table S3: Detailed information regarding the included literature**

*The included literature is presented in the assigned categories. Sources that are used in several categories are highlighted in blue.*

| Authors | Year | Study design | Sample size | Participants | Setting | Coun-try | Key finding |
|---|---|---|---|---|---|---|---|
| **Contraception** | | | | | | | |
| Shah, J.P.; Jatlaoui, T.C.; Zapata, L.B.; Curtis, K.M.; Pagano, H.P.; Whiteman, M.K. | 2019 | Prospective trial | 2,118 | physicians | Public-sector health centers | United States | Lack of knowledge regarding contraception after bariatric surgery in physicians |
| Damhof, M.A.; Pierik, E.; Krens, L.L.; Vermeer, M.; van Det, M.J.; van Roon, E.N. | 2019 | Prospective clinical trial | 230 | Female patients who underwent bariatric surgery | Clinic | The Nether-lands | Suboptimal contraceptive counseling and contraception |
| Graham, Y.N.H.; Mansour, D.J.; Small, P.K.; Fraser, I.S. | 2018 | Prospective clinical trial | 42 | Female patients who wait for bariatric surgery | Anonymous online survey | United King-dom | Sufficient use of safe contraceptive methods and high prevalence of menstrual disorders that might be associated to PCOS |
| Mengesha, B.M.; Carter, J.T.; Dehlendorf, C.E.; Rodriguez, A.J.; Steinauer, J.E. | 2018 | Prospective clinical trial | 363 | Females that have shown interest in bariatric surgery and weight loss | Online survey using face-book | United States | subjectively inadequate contraceptive counseling; postoperative safe contraception associated with perioperative contraceptive counseling |
| Mengesha, B.; Griffin, L.; Nagle, A.; Kiley, J. | 2016 | Retrospective chart survey | 1012 | Female patients who underwent bariatric surgery | Clinic | United States | Low rate of documented contraceptive counseling before surgery; pregnancy rate |
| Casas, R.; Bourjeily, G.; Vithiananthan, S.; Tong, I. | 2014 | Prospective clinical trial | 35 | Female patients who underwent bariatric surgery | Clinic | United States | postoperative safe contraception associated with perioperative contraceptive counseling |

| | | | | | | | |
|---|---|---|---|---|---|---|---|
| Michalsky, M.; Eneli, I.; April labuda; McCracken, K. | 2019 | Prospective clinical intervention study | 30 | Severely obese female adolescents | Clinic/ medical practice | United States | Improved contraception due to counseling in female adolescents |
| **Fetility/ Infertility** | | | | | | | |
| Menke, M.N., King, W.C., White, G.E., Gosman, G.G., Courcoulas, A.P., Dakin, G.F., Flum, D.R., Orcutt, M.J., Pomp, A., Pories, W.J., Purnell, J.Q., Steffen, K.J., Wolfe, B.M., Yanovski, S.Z. | 2019 | Prospective multicenter clinical trial | 640 | Female patients who underwent bariatric surgery | Clinic | United States | higher postoperative conception rate in nulliparous women with a preoperative history of infertility |
| Vincentelli, C., Maraninchi, M., Valéro, R., Béliard, S., Maurice, F., Emungania, O., Berthet, B., Lombard, E., Dutour, A., Gaborit, B., Courbiere, B. | 2018 | Prospective clinical trial | 39 | Female patients who underwent bariatric surgery | Clinic | France | decrease in the AMH concentration after bariatric surgery |
| Milone, M., Sosa Fernandez, L.M., Sosa Fernandez, L.V., Manigrasso, M., Elmore, U., De Palma, G.D., Musella, M., Milone, F. | 2017 | Retrospective chart survey | 40 | Female patients who underwent bariatric surgery | assisted reproductive technology center | Italy | improvement of the outcome of assisted reproductive technology as a result of bariatric surgery |
| Edison, E., Whyte, M., van Vlymen, J., Jones, S., Gatenby, P., de Lusignan, S., Shawe, J. | 2016 | Retrospective epidemiological survey | 16,295 | Female patients who underwent bariatric surgery | Clinic | United King-dom | bariatric surgery improves factors that underlie fertility and pregnancy outcomes |
| Al kabbi, M.S., Al-Taee, H.A., Al Hussaini, S.K. | 2018 | Prospective clinical trial | 60 | Female patients who underwent bariatric surgery | Clinic | Iraq | decrease in the AMH concentration after bariatric surgery |
| Soares Júnior, J.M., Lobel, A., Ejzenberg, D., Serafini, P.C., Baracat, E.C. | 2018 | Comment concerning previous surveys | - | - | - | Brazil | recommend to consider bariatric surgery as an alternative or even definitive solution for women suffering from morbid obesity and infertility |

| | | | | | | | |
|---|---|---|---|---|---|---|---|
| Ahmed, H.O. | 2017 | Prospective clinical trial | 167 | Female patients who underwent bariatric surgery | Clinic | Iraq | Improvement of fertility in obese females with PCOS postbariatric surgery |
| Chiofalo, F., Ciuoli, C., Formichi, C., Selmi, F., Forleo, R., Neri, O., Vuolo, G., Paffetti, P., Pacini, F. | 2017 | Prospective clinical trial | 55 | Obese women | Clinic | Italy | decrease in the AMH concentration after bariatric surgery |
| Bhandari, S., Ganguly, I., Bhandari, M., Agarwal, P., Singh, A., Gupta, N., Mishra, A. | 2016 | Prospective clinical trial | 75 | Female patients who underwent bariatric surgery | Clinic | India | decrease in the AMH concentration after bariatric surgery |
| Charalampakis, V., Tahrani, A.A., Helmy, A., Gupta, J.K., Singhal, R. | 2016 | Systematic literature review | - | - | - | United King-dom | preoperative infertility may be improved as a result of bariatric surgery |
| **Pelvic Floor Disorders and urinary incontinence** | | | | | | | |
| Keller, K.D.; Rosa, V.L.; Cerentini, T.M.; Souza, C.M. de; Costa, F.L.; Rosa, P.V. da; Klahr, P. da S.; Pereira, E. de A.; Rosa, L.T. | 2019 | Prospective clinical trial | 26 | Female patients with urinary incontinence who underwent bariatric surgery | Clinic | Brazil | Quality of life improvement after bariatric surgery, decreased severity of urinary incontinence |
| Paka, C.; Hallock, J.; Trock, B.; Steele, K.; Wright, E.J. | 2019 | Prospective clinical trial | 315 | Female patients who had shown interest in bariatric surgery | Anonymous survey | United States | Less knowledge about pelvic floor disorders in study population than general population |
| Zhang, J., Gao, L., Liu, M., & Liu, C. | 2018 | Systematic literature review | - | Survexs that include female patients who underwent bariatric surgery | - | China | Increased quality of life and improved function of pelvic floor disorders after bariatric surgery |

| | | | | | | | |
|---|---|---|---|---|---|---|---|
| Gabriel, I.; Tavakkoli, A.; Minassian, V. | 2018 | Prospective clinical trial | 447 | Female patients who underwent bariatric surgery | Clinic | United States | Inverse relationship between amount of weight loss and urinary incontinence and pelvic organ prolapse |
| Lian, W.; Zheng, Y.; Huang, H.; Chen, L.; Cao, B. | 2017 | Systematic quantitative review | 784 | Female patients who underwent bariatric surgery | Clinic | China | Postoperative improvement in urinary incontinence and pelvic organ prolapse, not in fecal incontinence and sexual function |
| Kim, J.H., Sun, H.Y., Lee, H.Y., Soh, M.J., Park, S., Kim, Y.J., Song, Y.S., 2017. Improvement of voiding characteristics in morbidly obese women after bariatric surgery: A single-center study with a 1-year follow-up. Surg. Obes. Relat. Dis. 13, 836–841. | 2017 | Prospective clinical trial | 57 | Female patients who underwent bariatric surgery | Clinic | Korea | Improvements in voiding status |
| O'Boyle, C.J., O'Sullivan, O.E., Shabana, H., Boyce, M., O'Reilly, B.A. | 2015 | Prospective clinical trial | 82 | Female patients who underwent bariatric surgery | Clinic | Ireland | Improvement of urinary incontinence, not a proportional relationship to preoperative BMI |
| Shimonov, M.; Groutz, A.; Schachter, P.; Gordon, D. | 2017 | Prospective clinical trial | 77 | Female patients who underwent bariatric surgery | Clinic | Israel | Reduction of symptoms of urinary incontinence |
| Anglim, B.; O'Boyle, C.J.; O'Sullivan, O.E.; O'Reilly, B.A. The long-term effects of bariatric surgery on female urinary incontinence. Eur. J. Obstet. Gynecol. Reprod. Biol. 2018, 231, 15–18. | 2018 | Prospective clinical trial | 366 | Females who underwent bariatric surgery | Clinic | Ireland | Long-term improvement of urinary incontinence |
| Leshem, A.; Shimonov, M.; Amir, H.; Gordon, D.; Groutz, A. | 2017 | Prospective clinical trial | 150 | Females who underwent bariatric surgery | Clinic | Israel | Improvement of pelvic floor disorders including colorectal-anal symptoms |

| | | | | | | | |
|---|---|---|---|---|---|---|---|
| Knepfler, T.; Valero, E.; Triki, E.; Chilintseva, N.; Koensgen, S.; Rohr, S | 2015 | Prospective clinical trial | 116 | Female patients who underwent bariatric surgery | Clinic | France | Improvement of pelvic floor disorders after surgery, high prevalence |
| Uruç, F., Akan, S., Aras, B., Şahin, A., Uruç, E., Yüksel, Ö.H., Ürkmez, A., Yıldırım, Ç. | 2016 | Prospective clinical trial | 53 | Female patients who underwent bariatric surgery | Clinic | Turkey | Reduction of symptoms of pelvic floor disorders |
| Al-Shaikh, G.K., Ibrahim, L., Al-Mandeel, H., Alshaikh, R., Syed, S.B. | 2018 | Prospective clinical trial | 166 | Obese Women awaiting bariatric surgery vs non-obese controls | Clinic | Saudia Arabia | Increased frequency of urinary incontinence and pelvic floor disorders in obese women |
| **Pregnancy and breast feeding** | | | | | | | |
| Maslin K, James A, Brown A, Bogaerts A, Shawe J. | 2019 | Systematic quantitative review | 202 | Female patients who underwent bariatric surgery | Clinic | United King-dom (Au-thors) | Lack of data concerning micronutrient intake |
| Bartholomay, L.M.; Berlin, K.; McInerney, M.; Garcia, L. | 2019 | Prospective clinical trial | 40 | Female patients undergoing bariatric surgery | Clinic | United States | More research is needed to determine suitable vitamin K measures |
| Balestrin, B.; Urbanetz, A.A.; Barbieri, M.M.; Paes, A.; Fujie, J. | 2019 | Retrospective chart survey | 298 | Women post bariatric surgery and obese pregnant women | Clinic | Brazil | Lower occurrence of health-related issues complicating pregnancy among women who had undergone bariatric surgery |
| Garretto, D.; Kim, Y.-K.; Quadro, L.; Rhodas, R.R.; Pimentel, V.; Crnosija, N.A.; Nie, L.; Bernstein, P.; Tropper, P.; Neal-Perry, G.S. | 2019 | Prospective clinical trial | 119 | Women after bariatric surgery and control patient | Clinic | United States | Nutritional deficiencies in breastfeeding women after bariatric surgeries may in fact be less common than in control women in an inner city |

| | | | | | | | |
|---|---|---|---|---|---|---|---|
| Faria, S.L.; Faria, O.P.; de Gouvêa, H.R.; Amato, A.A. | 2019 | Retrospective chart survey | 23 | Women after bariatric surgery | Clinic | Brazil | Satisfactory adherence to post-op micronutrient supplementation and few gestational complications following bariatric surgery |
| Harreiter, J.; Schindler, K.; Bancher-Todesca, D.; Göbl, C.; Langer, F.; Prager, G.; Gessl, A.; Leutner, M.; Ludvik, B.; Luger, A. | 2018 | Systematic literature review | - | Articles that include pregnant women after bariatric surgery | - | Austria (Authors) | Care of pregnant women after bariatric surgery needs to be organized in an individual setting in a multilateral cooperation of various medical disciplines in specialized centres |
| Jans, G.; Matthys, C.; Bogaerts, A.; Ameye, L.; Delaere, F.; Roelens, K.; Loccufier, A.; Logghe, H.; De Becker, B.; Verhaeghe, J.; et al. | 2018 | Prospective multicenter clinical trial | 79 | Women after bariatric surgery and obese women | Clinic | Belgium | Pregnancy following bariatric surgery induces high levels of anxiety that are not associated with an inadequate maternal diet |
| Hazart, J.; Le Guennec, D.; Accoceberry, M.; Lemery, D.; Mulliez, A.; Farigon, N.; Lahaye, C.; Miolanne-Debouit, M.; Boirie, Y. | 2017 | Retrospective chart survey | 48 | Women after bariatric surgery | Clinic | France | Prevalence of micronutritional deficiencies and small-for-gestational-age neonates is high in pregnant women following bariatric surgery |
| Gimenes, J.C.; Nicoletti, C.F.; de Souza Pinhel, M.A.; Cortes-Oliveira, C.; Salgado Júnior, W.; Nonino, C.B. | 2018 | Retrospective chart survey | 13 | Children from Women with Previously Bariatric Surgery | Clinic | Brazil | Children from women with previously gastric bypass presented low birth weight |
| Stuart, A.; Källen, K | 2017 | Epidemiological survey | 24452 | Women undergone bariatric surgery and control group | Clinic | Sweden | Bariatric surgery is associated with an increased risk of abdominal surgery during pregnancy |

| | | | | | | | |
|---|---|---|---|---|---|---|---|
| Dolin, C.; Ude Welcome, A.O.; Caughey, A.B. | 2016 | Systematic literature review | - | Surveys that include pregnant women who had undergone bariatric surgery | - | United States (Authors) | Pregnancy after bariatric surgery is safe and may be associated with improved pregnancy outcomes |
| Parent, B.; Martopullo, I.; Weiss, N.S.; Khandelwal, S.; Fay, E.E.; Rowhani-Rahbar, A. | 2017 | Retrospective chart survey | 10296 | Mothers with and without a history of bariatric surgery and their infants | Clinic | United States | Infants of mothers with a previous bariatric operation had a greater likelihood of perinatal complications |
| Neovius, M.; Stephansson, O. | 2016 | Comment concerning previous surveys | - | - | - | - | Request for careful monitoring of fetal growth and maternal nutritional status after bariatric surgery |
| Jans, G.; Matthys, C.; Bel, S.; Ameye, L.; Lannoo, M.; Van der Schueren, B.; Dillemans, B.; Lemmens, L.; Saey, J.-P.; van Nieuwenhove, Y.; et al. | 2016 | Prospective multicenter clinical trial | ongoing | Women before and after bariatric surgery | Clinic | Belgium | Monitoring women before undergoing bariatric surgery until a subsequent pregnancy and postpartum period |
| Edison, E.; Whyte, M.; van Vlymen, J.; Jones, S.; Gatenby, P.; de Lusignan, S.; Shawe, J. | 2016 | Retrospective chart survey | 16295 | Female population of reproductive age having bariatric surgery | National Bariatric Surgery Registry and Health Survey | United Kingdom | Bariatric surgery improves factors that underlie fertility and pregnancy outcomes |
| Abenhaim, H.A.; Alrowaily, N.; Czuzoj-Shulman, N.; Spence, A.R.; Klam, S.L. | 2016 | Retrospective chart survey | 23,1167 | Morbidly obese women and women with bariatric surgery | Nationwide Inpatient Sample | Canada | Women who undergo bariatric surgery have improved pregnancy outcomes as compared with morbidly obese women but bariatric surgery group was more likely to have complications |

| | | | | | | | |
|---|---|---|---|---|---|---|---|
| Kumari, A.; Nigam, A. | 2015 | Educational paper | - | Surveys that include women giving birth after bariatric surgery | - | India (Authors) | Pregnancy after bariatric surgery should be managed by team of experts involving the obstetrician, the bariatric surgeon and the nutritionist |
| Robson, S.; Daniels, B.; Rawlings, L. | 2016 | Comment concerning previous surveys | - | - | - | Australia (Authors) | Lack of evidence concerning bariatric treatment and pregnancy |
| Monshi, B.; Stockinger, T.; Vigl, K.; Richter, L.; Weihsengruber, F.; Rappersberger, K. | 2015 | Case report | 2 | Women with dermatological signs of combined post-gestational nutritional deficiencies following Roux-en-Y gastric bypass surgery | Clinic | Austria | Potentially increased risk of women to develop post-gestational dermatological manifestations of malnutrition following bariatric surgery |
| Parker MH, Berghella V, Nijjar JB. | 2016 | Retrospective chart survey | 18,6605 | Obese women with singleton gestations | Health care Cost and Utilization Project Nationwide Inpatient Sample | United States | Singleton gestations in obese women with a prior bariatric surgery have an increased risk of intrauterine growth restriction |
| Ciangura, C.; Coupaye, M.; Deruelle, P.; Gascoin, G.; Calabrese, D.; Cosson, E.; Ducarme, G.; Gaborit, B.; Lelièvre, B.; Mandelbrot, L.; | 2019 | Clinical practice guideline | - | - | - | France (Authors) | Clinical practice recommendations for pregnancy management following bariatric surgery |

| | | | | | | | |
|---|---|---|---|---|---|---|---|
| Abraham, J.; Neha, S.; Power, A | 2019 | Clinical practice guideline | - | - | - | United Kingdom (Authors) | Outlining the most common bariatric procedures and specific considerations, including nutritional supplementation required when providing care to women in the antenatal and postnatal period. |
| Newlan, L.; Geraghty, S. | 2018 | Systematic literature review | - | Pregnant Women Post Bariatric Surgery | - | United Kingdom (Authors) | A cost-effective alternative to an oral glucose tolerance test is to monitor pre-and post-meal blood glucose levels |
| Jans, G., Devlieger, R., Preter, V. D., Ameye, L., Roelens, K., Lannoo, M., Schueren, B. V. der, Verhaeghe, J., Matthys, C., De Preter, V., & Van der Schueren, B. | 2018 | Multicenter prospective clinical trial | ongoing | Women after bariatric surgery | Clinic | Belgium | Breast milk of women who have undergone BS appears to be adequate in energy, macronutrients, and vitamin A during the first 6 wk of lactation |
| Whyte, M.; Johnson, R.; Cooke, D.; Hart, K.; McCormack, M.; Shawe, J. | 2016 | Questionnaire-based online survey | 120 | Lead diabetes midwives | - | United Kingdom | Need for national clinical guidelines to be developed for the diagnosis of GDM after bariatric surgery |
| Galazis, N.; Docheva, N.; Simillis, C.; Nicolaides, K.H. | 2014 | Systematic literature review | 17 | Non-randomised cohort or case-control studies | - | United Kingdom (Authors) | Bariatric surgery as a whole improves some pregnancy outcomes |
| Maric, T.; Kanu, C.; Muller, D.; Tzoulaki, I.; Johnson, M.; Savvidou, M. | 2017 | prospective clinical trial | 129 | Pregnant women after bariatric surgery and without bariatric surgery | Clinic | United Kingdom | Birthweight was lower in the BS group compared to the no-surgery group |
| Opray, N.; Grivell, R.M.; Deussen, A.R.; Dodd, J.M. | 2015 | Systematic literature review | - | Studies that include overweight and obese women before pregnancy | - | Australia (Authors) | No findings of randomised controlled trials that assessed the effect of preconception health programs and interventions in overweight and obese women with the aim |

| | | | | | | | |
|---|---|---|---|---|---|---|---|
| | | | | | | | of improving pregnancy outcomes |
| Leclercq, W.K.G.; Van Sambeek, A.; Uittenbogaart, M.; Niemarkt, H.J.; Bongers, M.Y.; Van Laar, J.O.E.H. | 2018 | Case report | 2 | Pregnant women after laparoscopic gastric bypass | Clinic | The Netherlands | Presence of a multidisciplinary Bariatric-Obstetric-Neonatal team allows for an increased awareness of potential long-term complications associated with earlier bariatric surgery in pregnancy |
| **Female specific cancer** | | | | | | | |
| Ganesan, K., Choy, B.J.K. | 2019 | Comment concerning previous survey | - | - | - | United King-dom (Au-thors) | limitations in a previous study that surveyed the risk of developing breast cancer in the context of bariatric surgery |
| Feigelson, H., Caan, B., Weinmann, S., Leonard, A., Powers, J., Yenumula, P., Arterburn, D., Koebnick, C., Altaye, M., Schauer, D. | 2019 | Retrospective epidemiological survey | 71,887 | patients enrolled in an integrated health care delivery system | Clinic | United States | Bariatric surgery was associated with a reduced risk of breast cancer among severely obese women |
| Feigelson, H., Caan, B., Weinmann, S., Leonard, A., Powers, J., Yenumula, P., Arterburn, D., Koebnick, C., Altaye, M., Schauer, D. | 2019 | Answer to a comment concerning a previous publication | - | - | - | United States (Au-thors) | Availability of data |
| Neff, R., Havrilesky, L.J., Chino, J., O'Malley, D.M., Cohn, D.E. | 2015 | Economical simulation analyzis | 96,232 | Simulation patients: women with low risk stage I endometrioid EC | Statistical simulation | United States (Au-thors) | bariatric surgery appears to be a cost-effective intervention for women with low risk, early stage endometrial cancer |
| Sanguankeo, A., Upala, S | 2016 | Comment concerning previous survey | - | - | - | United States & Thailand (Au-thors) | gynecologists should strongly consider surgical weight loss as a therapeutic option in suitable endometrial cancer candidates |

| | | | | | | | |
|---|---|---|---|---|---|---|---|
| Modesitt, S.C., Hallowell, P.T., Slack-Davis, J.K., Michalek, R.D., Atkins, K.A., Kelley, S.L., Arapovic, S., Shupnik, M.A., Hoehn, K. | 2015 | prospective observational study | 75 | Female patients who underwent bariatric surgery | Clinic | United States | Bariatric surgery not only changed risk variables for the development of endometrial cancer but also improved the health-related quality of physical health significantly |
| Benito, V., López-Tomassetti, E., Esparza, M., Arencibia, O., Andújar, M., Prieto, M., Lubrano, A. | 2015 | Case report | 1 | Obese adolescent with endometrial carcinoma and initial failure of a fertility-preserving treatment | Clinic | Spain | After bariatric surgery and normalization of body weight, a patient with endometrial carcinoma showed improved response to the local treatment of the endometrial cancer |
| Anveden, Å., Taube, M., Peltonen, M., Jacobson, P., Andersson-Assarsson, J.C., Sjöholm, K., Svensson, P.-A., Carlsson, L.M.S | 2017 | Prospective epidemiological survey | 2867 | Obese women who obtained bariatric and non-bariatric treatment | Clinic | Sweden | Bariatric surgery was associated with reduced risk for female-specific cancer |
| Hoel, E. | 2015 | Literature review | - | - | - | United States (Authors) | research strengthen the causal link between obesity and cancer, specifically breast and endometrial cancers in women, and show that risk is reversible through bariatric surgery |
| Jernigan, A. m., Maurer, K., Cooper, K. r., Rose, P. g., Schauer, P. r., Michener, C. m. | 2016 | Prospective observational survey | 156 | Women with endometrial carcinoma or complex atypical hyperplasia and BMI > 30 kg/m² | Clinic | United States | The identification of surgery-specific aspects (concerning female-specific cancer) is difficult because non-surgery weight loss attempts usually do not offer a sufficient weight loss, at least on a long term basis. |

| | | | | | | | |
|---|---|---|---|---|---|---|---|
| Kitson, S., Ryan, N., MacKintosh, M.L., Edmondson, R., Duffy, J.M., Crosbie, E.J. | 2018 | Systematic quantitative review | 161 | Female bariatric patients of three RCTs | - (RCTs from Clinic) | United King-dom (Au-thors) | In contrast to the results of bariatric surgery, behavior and lifestyle interventions were not associated to an improved overall survival, nor a cancer-specific survival or an improved health-related quality of life |
| Fernandez-Montoli, M.-E., Sabadell, J., Martínez-García, J.M., Perez, N.A.C. | 2018 | Protocol | - | - | - | United King-dom (Au-thors) | Protocol for planned review |
| Upala, S., Sanguankeo, A. | 2016 | Systematic quantitative review | 890,110 | Female patients who underwent bariatric surgery | - (RCTs from Clinic) | United States (Au-thors) | reduced risk of endometrial cancer postoperatively |
| Charalampakis, V., Tahrani, A.A., Helmy, A., Gupta, J.K., Singhal, R. (see above) | 2016 | Literature review | - | - | - | United King-dom (Au-thors) | preoperative infertility may be improved as a result of bariatric surgery & recommendation to offer bariatric surgery to severely obese patients who suffer from obesity-associated gonadal dysfunction |
| Needleman, B.J., Noria, S.F. | 2015 | Comment concerning previous research | - | - | - | United States (Au-thors) | bariatric surgery has a positive effect on endometrial hyperplasia making surgically induced weight loss a potentially attractive option for endometrial cancer prevention and treatment |
| Wee, C.C., Davis, R.B., Jones, D.B., Apovian, C.A., Chiodi, S., Huskey, K.W., Hamel, M.B. | 2016 | Prospective observational survey | 574 | Patients seeking bariatric surgery | Clinic | United States | Quality of life varied across race/ethnicity and sex |

| | | | | | | | |
|---|---|---|---|---|---|---|---|
| **Menopause and osteoporosis** | | | | | | | |
| Luhrs, A.R., Davalos, G., Lerebours, R., Yoo, J., Park, C., Tabone, L., Omotosho, P., Torquati, A., Portenier, D., Guerron, A.D. | 2019 | prospective clinical trial | 28 | patients who underwent bariatric surgery | Clinic | United States | Deterioration of bone quality after bariatric surgery, independent of the bariatric procedure |
| Goughnour, S.L., Thurston, R.C., Althouse, A.D., Freese, K.E., Edwards, R.P., Hamad, G.G., McCloskey, C., Ramanathan, R., Bovbjerg, D.H., Linkov, F. | 2016 | prospective clinical trial | 69 | patients who underwent bariatric surgery | Clinic | United States | bothersome menopausal symptoms may become rarer after bariatric surgery |
| Menegati, G.C., de Oliveira, L.C., Santos, A.L.A., Cohen, L., Mattos, F., Mendonça, L.M.C., Carneiro, J.R.I., Farias, M.L.F., Rosado, E.L. | 2016 | Clinical trial | 58 | premenopausal women who had undergone RYGBP and matched control group | Clinic | Brazil | Vitamin D deficiency may appear in that later postoperative period |
| Sánchez, A., Rojas, P., Basfi-fer, K., Carrasco, F., Inostroza, J., Codoceo, J., Valencia, A., Papapietro, K., Csendes, A., Ruz, M. | 2016 | Clinical trial | 103 | Patients seeking bariatric surgery | Clinic | Chile | High prevalence for micronutrient deficiency |
| Casimiro, I., Sam, S., Brady, M.J. | 2019 | Literature review | - | - | - | United States (Authors) | bariatric surgery may result in bone loss due to potential vitamin D deficiency, mechanical unloading from weight loss, and changed hormonal secretion such as a reduction in leptin, and estrogen |
| **Sexuality and sexual function** | | | | | | | |
| Pichlerova, D., Bob, P., Zmolikova, J., Herlesova, J., Ptacek, R., Laker, M.K., Raboch, J., Fait, T., Weiss, P. | 2019 | Questionnaire-based clinical trial | 120 | Female patients who underwent bariatric surgery and control group | Clinic | Czech Republic | presence of obesity is correlated to sexual and erectile dysfunction |

| | | | | | | | |
|---|---|---|---|---|---|---|---|
| Steffen, K.J., King, W.C., White, G.E., Subak, L.L., Mitchell, J.E., Courcoulas, A.P., Flum, D.R., Strain, G., Sarwer, D.B., Kolotkin, R.L., Pories, W., Huang, A.J. | 2019 | Questionnaire-based data collection in a multicenter cohort study | 1,751 | Female patients who underwent bariatric surgery | Clinic | United States | women who were dissatisfied with their sexual life reported a meaningful improvement after bariatric surgery in the follow up |
| Lechmiannandan, S., Panirselvam, M., Muninathan, P., Hussin, N., Rajan, R., Sidi, H., Kosai, N.R., Vinayak, C.R. | 2019 | Prospective observational survey | 52 | Female patients who underwent bariatric surgery | Clinic | Malaysia | Female sexual dysfunction is highly prevalent among the obese women and may benefit from bariatric surgery |
| Sarwer, D.B., Wadden, T.A., Spitzer, J.C., Mitchell, J.E., Lancaster, K., Courcoulas, A., Gourash, W., Rosen, R.C., Christian, N.J. | 2018 | Prospective clinical trial | 106 | Female patients who underwent bariatric surgery | Clinic | United States | Improvements in reproductive hormones and physical aspects of QOL, body image, and depressive symptoms persisted but improvements in sexual functioning, relationship satisfaction, and mental components of QOL eroded over time after bariatric surgery |
| Steffen, K.J., King, W.C., White, G.E., Subak, L.L., Mitchell, J.E., Courcoulas, A.P., Flum, D.R., Strain, G., Sarwer, D.B., Kolotkin, R.L., Pories, W., Huang, A. | 2017 | Questionnaire-based data collection in a multicenter cohort study | 1,751 | Female patients who underwent bariatric surgery | Clinic | United States | High prevalence of limitation of sexual activity due to physical health in and sexual dissatisfaction in severely obese women |
| Ramalho, S., Bastos, A.P., Silva, C., Vaz, A.R., Brandão, I., Machado, P.P.P., Conceição, E. | 2015 | Interview-based clinical trial | 61 | Female patients who had undergone bariatric surgery | Clinic | Portugal | sexual intimacy and body image should be considered in the bariatric treatment in order to optimize psychological and weight outcomes after bariatric surgery |

| | | | | | | | |
|---|---|---|---|---|---|---|---|
| Janik, M.R., Bielecka, I., Paśnik, K., Kwiatkowski, A., Podgórska, L. | 2015 | Questionnaire-based cohort survey | 153 | Female patients who had undergone bariatric surgery | Online data collection | Poland | prevalence of female sexual dysfunction did not change significantly but the sexual quality of life was significantly higher in the follow-up compared to the preoperative value |
| Zeller, M.H., Brown, J.L., Reiter-Purtill, J., Sarwer, D.B., Black, L., Jenkins, T.M., McCracken, K.A., Courcoulas, A.P., Inge, T.H., Noll, J.G., Doland, F., Morgenthal, A., Howarth, T., Comstock, S., Kirk, S., Helmrath, M., Lee, M.C., Allen, D., Garland, B., Washington, G., Mikhail, C., Brandt, M.L., Blake, R., El Nokali, N., Rofey, D., Arslanian, S., Desai, K., Seay, A., Haynes, B., Austin, H., Harmon, C., Ginn, M., Baughcum, A.E., Michalsky, M.P., Christian, M.S., Andringa, J., Powers, C., Akers, R., TeenView, S.G., Teen-LABS, C. | 2019 | Prospective multicenter clinical trial | 111 | Female adolescents with severe obesity | Clinic | United States | greater increase in behaviours that pose a risk of sexually transmitted infections, early motherhoods and pregnancies in females that underwent bariatric surgery |
| Oliveira, C.F. de A., dos Santos, P.O., de Oliveira, R.A., Leite-Filho, H., de Almeida Oliveira, A.F., Bagano, G.O., Lima Junior, E.B., Miranda, E.P., de Bessa Junior, J., Barroso Junior, U. | 2018 | Prospective clinical trial | 62 | Female patients undergoing bariatric surgery | Clinic | Brazil | melioration of several sex positions after bariatric surgery |
| Cherick, F., Te, V., Anty, R., Turchi, L., Benoit, M., Schiavo, L., Iannelli, A. | 2019 | Prospective clinical trial | 43 | Female patients undergoing bariatric surgery | Clinic | France | amelioration of female sexual dysfunction 12 months after bariatric surgery |

| | | | | | | | |
|---|---|---|---|---|---|---|---|
| **Sex hormons and PCOS** | | | | | | | |
| Abiad, F., Khalife, D., Safadi, B., Alami, R., Awwad, J., Khalifeh, F., Ghazeeri, G. | 2018 | Prospective clinical trial | 44 | Female patients undergoing bariatric surgery | Clinic | Lebanon | The degree of weight loss after surgery was effective in lowering CRP and increasing adiponectin levels in PCOS women but lower than in those without PCOS. |
| Christ, J., Falcone, T | 2018 | Retrospective chart survey | 44 | Female patients who underwent bariatric surgery | Clinic documentation | United States | reduction of ovarian volume after bariatric surgery in severely obese women suffering PCOS |
| Sarwer, D.B., Wadden, T.A., Spitzer, J.C., Mitchell, J.E., Lancaster, K., Courcoulas, A., Gourash, W., Rosen, R.C., Christian, N.J. | 2018 | Prospective clinical trial | 106 | Female patients undergoing bariatric surgery | Clinic | United States | Persistence of hormonal changes 4 years after bariatric surgery |
| Escobar-Morreale, H.F., Santacruz, E., Luque-Ramírez, M., Botella Carretero, J.I. | 2017 | Systematic quantitative review | 382 | Female patients who underwent bariatric surgery | - (RCTs from Clinic) | Spain (Au-thors) | Since SHBG level increases while estradiol decreases after bariatric surgery, inhibitory influences arising from adipose tissue may dominate the picture in severe obesity |
| Bhandari, S., Ganguly, I., Bhandari, M., Agarwal, P., Singh, A., Gupta, N., Mishra, A. | 2016 | prospective observational study | 75 | Female patients who underwent bariatric surgery | Clinic | India | normalization of menstrual irregularities in PCOS- patients after bariatric surgery |
| Charalampakis, V., Tahrani, A.A., Helmy, A., Gupta, J.K., Singhal, R. (see above) | 2016 | Systematic literature review | - | - | - | United King-dom (Au-thors) | preoperative infertility may be improved as a result of bariatric surgery & recommendation to offer bariatric surgery to severely obese patients who suffer from obesity-associated gonadal dysfunction |

| | | | | | | | |
|---|---|---|---|---|---|---|---|
| Casimiro, I., Sam, S., Brady, M.J. (see above) | 2019 | Systematic literature review | - | - | - | United States (Authors) | bariatric surgery may result in bone loss due to potential vitamin D deficiency, mechanical unloading from weight loss, and changed hormonal secretion such as a reduction in leptin, and estrogen |
| Christ, J., Falcone, T. | 2016 | Retrospective chart survey | 44 | Female patients who underwent bariatric surgery | Clinic documentation | United States | reduction of androgen levels and proportion of women who fulfill the criteria for hyperandrogenism and irregular menses after bariatric surgery |
| Graham, Y.N.H.; Mansour, D.J.; Small, P.K.; Fraser, I.S. (see above) | 2018 | prospective observational study | 42 | Female patients who wait for bariatric surgery | Anonymous online survey | United Kingdom | Sufficient use of safe contraceptive methods and high prevalence of menstrual disorders that might be associated to PCOS |
| **Metabolism, Outcome/ Follow-Up / quality of life** | | | | | | | |
| Kennedy-Dalby, A., Adam, S., Ammori, B.J., Syed, A.A. | 2014 | Prospective clinical trial | 158 | Patients undergoing bariatric surgery | Clinic | United Kingdom | no significant differences in the outcome regarding weight loss and improvement of obesity-related comorbidities between men and women |
| Young, M.T., Phelan, M.J., Nguyen, N.T. | 2016 | Nationwide epidemiological survey | 810,999 | Patients undergoing bariatric surgery | Clinic | United States | Men undergoing bariatric surgery tend to have higher severity of illness, with higher risk-adjusted serious morbidity and mortality rates |
| Afarideh, M., Ghajar, A., Nikdad, M.S., Alibakhshi, A. | 2016 | Comment concerning previous research | - | - | - | Iran (Authors) | Higher proportion of females among bariatric patients |
| Kochkodan, J., Telem, D.A., Ghaferi, A.A. | 2018 | Epidemiological survey | 61,708 | Patients undergoing bariatric surgery | Clinic | United States | males were significantly more satisfied with their operation |

| | | | | | | | |
|---|---|---|---|---|---|---|---|
| Keller, K.D., Rosa, V.L., Cerentini, T.M., Souza, C.M. de, Costa, F.L., Rosa, P.V. da, Klahr, P. da S., Pereira, E. de A., Rosa, L.T. (see above) | 2019 | Prospective clinical trial | 26 | Female patients with urinary incontinence who underwent bariatric surgery | Clinic | Brazil | improvement of urinary incontinence and quality of life after bariatric surgery |
| Grayson, B.E., Gutierrez-Aguilar, R., Sorrell, J.E., Matter, E.K., Adams, M.R., Howles, P., Karns, R., Seeley, R.J., Sandoval, D.A. | 2017 | Animal model survey | 52 | Rodents | Laboratory | United States | important sex-specific differences in the hepatic lipid metabolism after sleeve gastrectomy |
| Stroh, C., Weiner, R., Wolff, S., Knoll, C., de Zwaan, M., Manger, T. | 2015 | Epidemiological survey | 30,824 | Bariatric procedures | Clinic | Germany | Higher proportion of females in bariatric procedures |
| **Else** | | | | | | | |
| Horvath, C.M., Jossen, J., Kröll, D., Nett, P.C., Baty, F., Brill, A.-K., Ott, S.R. | 2018 | Prospective clinical trial | 251 | Patients undergoing bariatric surgery | Clinic | Switzerland | gender-specific difference in the performance of the evaluated obstructive sleep apnea screening questionnaires in patients scheduled for bariatric surgery |
| Biörserud, C., Shams, K., Elander, A., Olsén, M.F. | 2018 | Clinical trial | 131 | Patients who had undergone bariatric surgery | Clinic | Sweden | no differences in self-image or quality of life between the genders |
| Ahmed, H.O., Arif, S.H., Abdulhakim, S.A., Kakarash, A., Ali Omer, M.A., Nuri, A.M., Omer, H.H., Jalal, H.K., Omer, S.H., Muhammad, N.A | 2018 | Prospective clinical trial | 209 | Patients undergoing bariatric surgery | Clinic | Iraq | Improvement of fertility in obese females with PCOS after bariatric surgery |

**Table S4: Criteria for Literature search and inclusion**

| | | |
|---|---|---|
| **Literature search** | | |
| Language | | Article published in English |
| | | Summary or Abstract in English |
| Literature format | | - Journal literature<br>- Original research articles<br>- Case reports<br>- Review articles<br>- Comments<br>- Corrections<br>- Letter to the editor |
| | | Abstracts of recently published posters |
| **Inclusion criteria** | | |
| Content requirements | | Focus on bariatric treatment of women AND |
| | | Focus on gender- or sex-specific aspects |
| Journal requirements | | Free availability of an English abstract |
| | | Abstract provides information about the content of the article, e.g. type of the survey or review format |
| | | Publication in a journal with available ISSN |
| **Format-specififc requirements** | | |
| | Original research articles | All included; mentioning limitations, if necessary |
| | Systematic reviews | All included; mentioning limitations, if necessary |
| | Educational papers (non-systematic reviews) | Included, named as such |
| | Case reports | Included, named as such |
| | Comments | Included if referring to an included original research article/ systematic review and named as such |
| | Corrections | Included if referring to an included original research article/ systematic review and named as such |
| | Letter to the editor | Included if referring to an included original research article/ systematic review and named as such |
| | Abstracts of recently published posters | Included, if results of RCTs/ systematic reviews are presented |
